# Functionalized Hydroperoxide Formation from the Reaction of Methacrolein-Oxide, an Isoprene-Derived Criegee Intermediate, with Formic Acid: Experiment and Theory

**DOI:** 10.3390/molecules26103058

**Published:** 2021-05-20

**Authors:** Michael F. Vansco, Kristen Zuraski, Frank A. F. Winiberg, Kendrew Au, Nisalak Trongsiriwat, Patrick J. Walsh, David L. Osborn, Carl J. Percival, Stephen J. Klippenstein, Craig A. Taatjes, Marsha I. Lester, Rebecca L. Caravan

**Affiliations:** 1 Department of Chemistry, University of Pennsylvania, Philadelphia, PA 19104-6323, USA; vanscomf@anl.gov (M.F.V.); nisalak@sas.upenn.edu (N.T.); pwalsh@sas.upenn.edu (P.J.W.); 2 Argonne National Laboratory, Chemical Sciences and Engineering Division, Lemont, IL 60439, USA; sjk@anl.gov; 3 NASA Postdoctoral Program Fellow, NASA Jet Propulsion Laboratory, California Institute of Technology, 4800 Oak Grove Drive, Pasadena, CA 91109, USA; kristen.zuraski@noaa.gov; 4 NASA Jet Propulsion Laboratory, California Institute of Technology, 4800 Oak Grove Drive, Pasadena, CA 91109, USA; fred.a.winiberg@jpl.nasa.gov (F.A.F.W.); carl.j.percival@jpl.nasa.gov (C.J.P.); 5 Division of Chemistry and Chemical Engineering, California Institute of Technology, Pasadena, CA 91125, USA; 6 Combustion Research Facility, Mailstop 9055, Sandia National Laboratories, Livermore, CA 94551, USA; kau@sandia.gov (K.A.); dlosbor@sandia.gov (D.L.O.); 7 Department of Chemical Engineering, University of California, Davis, CA 95616, USA

**Keywords:** reaction intermediates, oxidation reactions, reaction pathways, kinetics, mass spectrometry, ionization, volatile organic compounds, atmospheric chemistry

## Abstract

Methacrolein oxide (MACR-oxide) is a four-carbon, resonance-stabilized Criegee intermediate produced from isoprene ozonolysis, yet its reactivity is not well understood. This study identifies the functionalized hydroperoxide species, 1-hydroperoxy-2-methylallyl formate (HPMAF), generated from the reaction of MACR-oxide with formic acid using multiplexed photoionization mass spectrometry (MPIMS, 298 K = 25 °C, 10 torr = 13.3 hPa). Electronic structure calculations indicate the reaction proceeds via an energetically favorable 1,4-addition mechanism. The formation of HPMAF is observed by the rapid appearance of a fragment ion at *m*/*z* 99, consistent with the proposed mechanism and characteristic loss of HO_2_ upon photoionization of functional hydroperoxides. The identification of HPMAF is confirmed by comparison of the appearance energy of the fragment ion with theoretical predictions of its photoionization threshold. The results are compared to analogous studies on the reaction of formic acid with methyl vinyl ketone oxide (MVK-oxide), the other four-carbon Criegee intermediate in isoprene ozonolysis.

## 1. Introduction

**Scheme 1:**
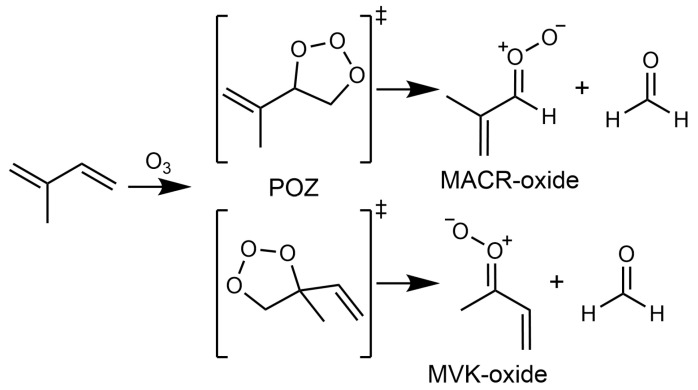
Reaction scheme illustrating the generation of *anti*-MACR-oxide + formaldehyde and *syn*-MVK-oxide + formaldehyde from the ozonolysis of isoprene.

Isoprene (2-methyl-1,3-butadiene), a five-carbon doubly unsaturated hydrocarbon, is the most abundant non-methane species emitted into Earth’s atmosphere. The primary source of isoprene is foliar emissions from the southern (48%) and northern (38%) tropics, with total emissions approaching 600 Tg year^−1^ [1]. Ozonolysis is an important sink of tropospheric isoprene (~10%) and proceeds via 1,3-cycloaddition of ozone to either of the two C=C double bonds to yield a highly internally excited primary ozonide (POZ) as shown in Scheme 1 [2,3].

The POZ subsequently decomposes to form carbonyl and zwitterionic carbonyl oxide products, the latter known as a Criegee intermediate [2,3]. Four pairs of reaction products are possible depending on which double bond of isoprene ozone adds to and the nature of the POZ decomposition. The four sets of products and relative yields from isoprene ozonolysis are formaldehyde oxide (CH_2_OO) + methyl vinyl ketone (ca. 17%), CH_2_OO + methacrolein (ca. 41%), methyl vinyl ketone oxide (MVK-oxide, CH_3_C(OO)CH(CH_2_) + formaldehyde (ca. 23%), and methacrolein oxide (MACR-oxide, HC(OO)C(CH_2_)CH_3_) + formaldehyde (ca. 19%) [2,4]. An illustration of the formation of MACR-oxide and MVK-oxide from isoprene ozonolysis is shown in Scheme 1.

The Criegee intermediates generated from isoprene ozonolysis are chemically activated and can undergo rapid unimolecular decomposition or can be thermalized through collisions to form stabilized Criegee intermediates [5]. Unimolecular decomposition of initially energized and thermalized Criegee intermediates strongly impacts the oxidizing capacity of the atmosphere by contributing to the daytime hydroxyl (OH) radical budget and essentially all of the OH radicals at nighttime [6,7]. Stabilized Criegee intermediates can further impact the oxidizing capacity of the atmosphere by acting as oxidants themselves [8,9,10,11], and undergo rapid reaction with tropospheric pollutants such as formic acid and SO_2_ [12,13,14,15,16]. The products from these reactions are implicated in the formation of secondary organic aerosol (SOA) that are known to impact urban visibility [17], human health [18,19], and global climate [20,21]. Reaction with formic acid generates highly oxygenated, lower-volatility, functionalized hydroperoxides that may be precursors to SOA [12,15,16], whereas reaction with SO_2_ forms SO_3_—a critical sulfuric acid precursor that results in sulfate aerosol production [22,23,24,25]. This study is comprised of an experimental and theoretical investigation of the reaction of thermalized MACR-oxide with formic acid and includes a direct comparison to the reaction of MVK-oxide with formic acid, demonstrating the differences in reactivity of these important atmospheric intermediates and atmospheric implications.

MACR-oxide and MVK-oxide are isomers of each other, differing only by the position of a methyl group. They differ from alkyl substituted Criegee intermediates in the presence of resonance stabilization through their conjugated pi bonds [12,26,27]. Each is predicted to have four conformational forms (*syn/anti-cis/trans*) with similar ground state energies (within ca. 3 kcal mol^−1^) compared to their respective lowest energy conformer [27,28]. The *syn* and *anti* conformers are separated by a high barrier (ca. 30 kcal mol^−1^) [28] for rotation about the C=O bond, such that interconversion is negligible at 298 K. Each *syn* and *anti* conformer comprises two configurations (*cis/trans*) that are distinguished by the orientation of the vinyl group with respect to the C=O bond. The *cis* and *trans* conformational forms are expected to rapidly interconvert at 298 K by rotation about the C–C bond (<10 kcal mol^−1^ barrier) resulting in an equilibrium mixture of *cis*/*trans* conformers [12].

Until recently, no synthetic methods for the isolated production of MACR-oxide and MVK-oxide were known, preventing the direct study of their reactivity. Lester and coworkers have demonstrated a photolytic method for selective production of MACR-oxide and MVK-oxide in laboratory experiments [27,28]. This alternate synthetic mechanism enabled the study of the electronic spectroscopy [26,27], infrared spectroscopy [28,29,30], unimolecular reactivity [28,29,31,32], and bimolecular reactivity [12,33,34,35] of the four-carbon isoprene-derived Criegee intermediates.

Criegee intermediates have been predicted and observed to have vast differences in their unimolecular and bimolecular reactivity depending on their substituents and conformational form [9,11,36]. The extended conjugation present between the carbonyl oxide moiety and vinyl group of the four-carbon isoprene derived Criegee intermediates fundamentally changes their electronic properties compared to Criegee intermediates with saturated alkyl substituents, altering their unimolecular and bimolecular reactivity [36]. The available unimolecular decay mechanisms, transition state (TS) barriers for unimolecular and bimolecular processes, and resultant rate coefficients are each impacted by the extended conjugation and the relative changes in these properties alters the fate of these Criegee intermediates in the atmosphere [28,37,38,39,40].

A novel unimolecular decay mechanism has been identified for some Criegee intermediates that have extended conjugation [38]. This mechanism is available for conformations in which the terminal oxygen is oriented toward the vinyl group (e.g., *syn*-MACR-oxide and *anti*-MVK-oxide) and proceeds via rapid 1,5 electrocyclic ring closure between the carbonyl oxide moiety and vinyl group to form 5-membered cyclic peroxides known as dioxoles [28,31,37,38]. The rapid unimolecular decay of these conformers (2500 s^−1^ for *syn*-MACR-oxide and 2140 s^−1^ for *anti*-MVK-oxide) suggests the lifetimes of *syn*-MACR-oxide and *anti*-MVK-oxide in the atmosphere are <1 ms, and bimolecular reactions, even those with large rate coefficients, cannot significantly compete for their removal [36]. In contrast, *syn*-MVK-oxide and *anti*-MACR-oxide are predicted to undergo unimolecular decay via pathways with higher transition state barriers, resulting in slow thermal decay rates (33 and 10 s^−1^, respectively, at 298 K = 25 °C and 760 torr = 101.3 kPa) [28,38]. For example, the unimolecular decay of *syn*-MVK-oxide has been shown to proceed through a 1,4 H-atom transfer at a rate slower than Criegee intermediates with saturated alkyl substituents that undergo an analogous unimolecular decay mechanism due to a higher transition state barrier. The larger transition state barrier is attributed to loss of the extended conjugation of *syn*-MVK-oxide [28,29,36,41]. For these conformers, unimolecular decay is sufficiently slow that some bimolecular reactions can compete for their removal.

The transition state barriers of bimolecular reactions involving MACR-oxide and MVK-oxide are also impacted by the disruption of their extended conjugation, resulting in higher transition state barriers for reaction [12,34,38,42,43]. Reactions of MACR-oxide and MVK-oxide with transition state barriers comparable to the energy of reactants (such as reactions with water vapor and alcohols [38,42,43]) are most affected. For example, the reactions of water vapor with *syn*-MVK-oxide and *anti*-MACR-oxide were found to be substantially slower than found for alkyl-substituted Criegee intermediates with similar carbon backbones and conformational forms that lack resonance stabilization [12,34]. In contrast, the rate coefficients for reaction of *syn*-MVK-oxide with formic acid and SO_2_ are as large as those for Criegee intermediates such as CH_2_OO [12,14,44,45]. While the barriers for these reactions are comparatively higher than for CH_2_OO, they are strongly submerged relative to reactants such that the rate coefficients for reaction are not greatly impacted. However, it has recently been shown that the reactivity of *anti*-MACR-oxide with SO_2_ is four times faster than that for *syn*-MVK-oxide, with rate coefficients of (1.5 ± 0.4) × 10^−10^ cm^3^ s^−1^ and (4.2 ± 0.6) × 10^−11^ cm^3^ s^−1^, respectively [12,34]. In addition, MACR-oxide was found to have a measurable, albeit slow, reaction with water vapor, whereas the reaction of MVK-oxide with water vapor is negligible [12,34]. This comparison suggests that even though disruption of the extended conjugation of MVK-oxide and MACR-oxide plays a significant role in dictating their reactivity, their structural differences also affect their reactivity.

We report herein the formation of a functionalized hydroperoxide species, 1-hydroperoxy-2-methylallyl formate (HPMAF), from the reaction of MACR-oxide with formic acid using multiplexed photoionization mass spectrometry (MPIMS, 298 K = 25 °C, 10 torr = 13.3 hPa). Complementary high-level ab initio calculations support the formation of HPMAF from this reaction via a nearly barrierless reaction pathway. A direct comparison is made to the reaction of MVK-oxide with formic acid to highlight how differences in hydrogen bonding affect reactions of the two isomers with formic acid.

## 2. Results and Discussion

**Scheme 2:**
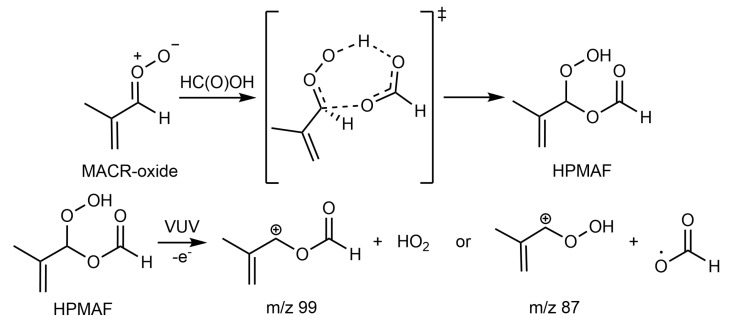
Mechanism for the reaction of MACR-oxide with formic acid, leading to the formation of 1-hydroperoxy-2-methylallyl formate (HPMAF). HPMAF is predicted to fragment upon VUV ionization into fragment ions at *m*/*z* 87 or 99. The calculated appearance energy of the *m*/*z* 87 and 99 fragment ion is 10.53 and 9.89 eV, respectively (CCSD(T)-F12/cc-pVTZ-F12//B2PLYP-D3/cc-pVTZ).

The reaction of MACR-oxide with formic acid is expected to generate 1-hydroperoxy-2-methylallyl formate (HPMAF), a functionalized hydroperoxide species, via a 1,4-addition mechanism (Scheme 2) analogous to the reaction of saturated Criegee intermediates and MVK-oxide with formic acid [12,15,16,46].

**Figure 1:**
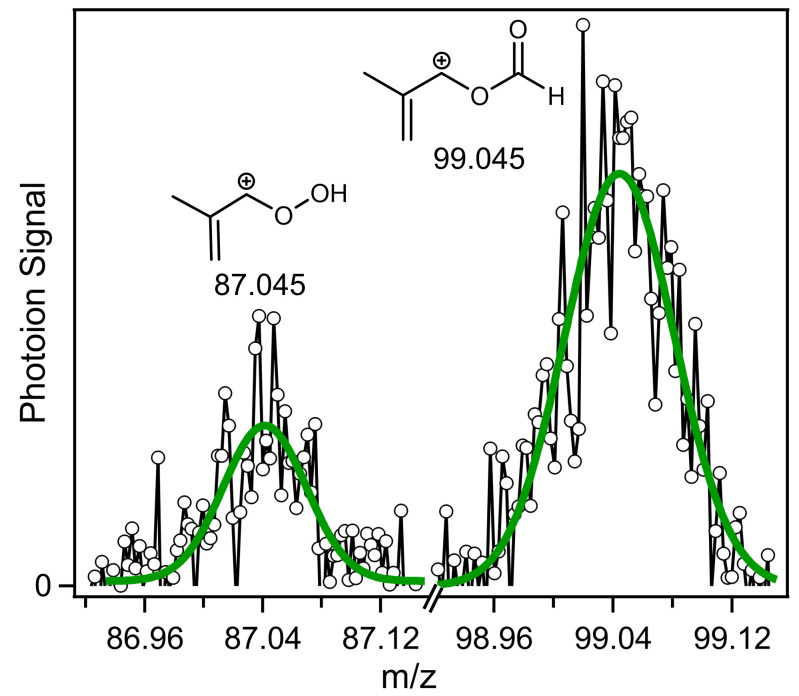
Fragment ions observed in the mass spectrum following the reaction of MACR-oxide with formic acid (2.6 × 10^13^ cm^−3^) using MPIMS. The mass spectrum is obtained by integrating over the full kinetic time window (0–60 ms) and VUV photon energy (9.0–11.0 eV). Gaussian fits to the mass peaks yield exact masses of 87.043 ± 0.003 and 99.045 ± 0.002, consistent with the chemical composition of C_4_H_7_O_2_ (87.045) and C_5_H_7_O_2_ (99.045) corresponding to HCO_2_-loss and HO_2_-loss from photoionization of HPMAF, respectively.

**Figure 2:**
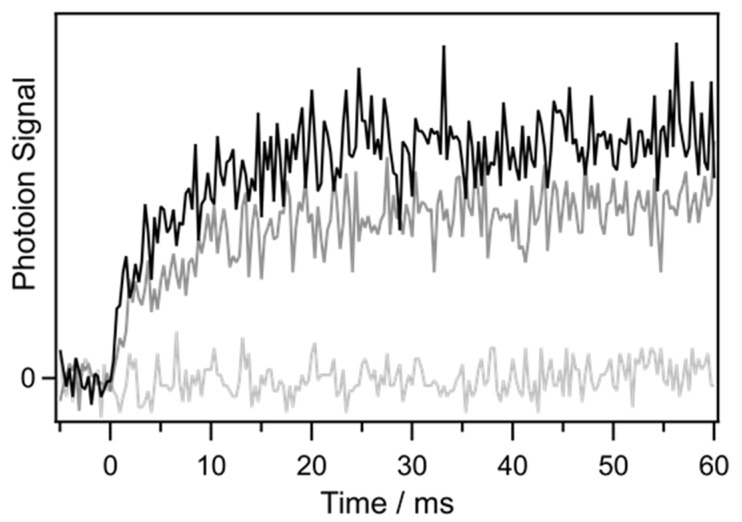
Temporal profile of *m*/*z* 99 observed from the reaction of MACR-oxide with formic acid (light grey line (formic acid) = 0 cm^−3^, dark grey line (formic acid) = 6.6 × 10^12^ cm^−3^, black line (formic acid) = 5.7 × 10^13^ cm^−3^) at a photoionization energy of 10.5 eV. The temporal profile reveals the rapid formation of a stable product that fragments upon ionization into a fragment ion at *m*/*z* 99. The small increase in the amplitude of the *m*/*z* 99 signal between the dark grey and black traces indicates the reaction is rapid and most of the MACR-oxide generated is reacted at (formic acid) = 6.6 × 10^12^ cm^−3^.

The proposed mechanism with formation of HPMAF is investigated via MPIMS experiments at 298 K (=25 °C) and 10 Torr (=13.3 hPa). Kinetically resolved signal is not observed on the parent mass channel of HPMAF (*m*/*z* 132) in the presence of formic acid. However, a strong signal appears in the mass spectrum at *m*/*z* 99. In addition, a weak signal present at *m*/*z* 87 is found to increase upon the addition of formic acid. Figure 1 shows a representative mass spectrum integrated over the full kinetic time window (0–60 ms) and VUV photon energy (9.0–11.0 eV, 50 meV steps). Gaussian fits of the mass spectra yield peak positions of 87.043 ± 0.003 and 99.045 ± 0.002, consistent with the exact chemical composition of C_4_H_7_O_2_ (87.045) and C_5_H_7_O_2_ (99.045), respectively. These product signals are in accord with HCO_2_-loss (*m*/*z* 87) and HO_2_-loss (*m*/*z* 99) fragment ions, respectively, observed from the photoionization of the functionalized hydroperoxide species formed from the reaction of MVK-oxide with formic acid (Scheme 1) [12,33]. The time profile of *m*/*z* 99 (Figure 2) obtained using 10.5 eV VUV photon energy exhibits a fast rise consistent with rapid reaction of Criegee intermediates with organic acids [12,16,33]. In addition, the amplitude of the product signal increases as a function of formic acid concentration indicating that it originates from reaction with MACR-oxide. The minor fragmentation pathway observed at *m*/*z* 87 is discussed in Section S1 of the Supplementary Materials (Figures S1–S3).

**Figure 3:**
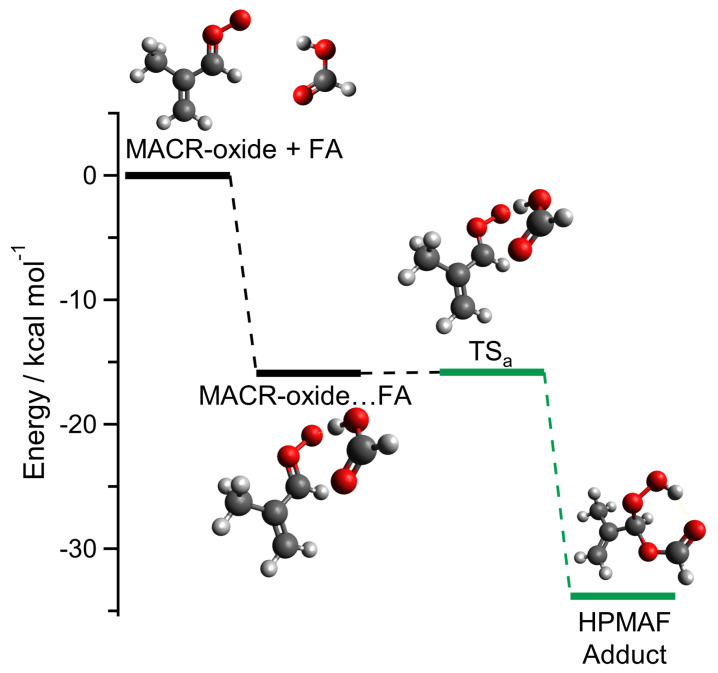
Reaction coordinate showing HPMAF formation from the 1,4-addition reaction of *anti*-*trans*-MACR-oxide with formic acid (FA, green) at the CCSD(T)-F12/cc-pVTZ-F12//B2PLYP-D3/cc-pVTZ level of theory including estimated CCSDT(Q) corrections.

Identification of HPMAF is supported by high-level ab initio calculations of the 1,4-addition reaction of *anti*-MACR-oxide with formic acid (Figure 3). The reaction begins with barrierless formation of a 7-membered cyclic pre-reactive complex (MACR-oxide…FA) that is substantially submerged (−15.9 kcal mol^−1^) relative to reactants. Rapid interconversion between *cis* and *trans* conformational forms of MACR-oxide within the pre-reactive complex structure is expected due to a low torsional barrier associated with the submerged transition state [33]. The transition state associated with the 1,4-addition reaction (TS_a_, −15.8 kcal mol^−1^) has a similar structure and nearly identical energy as the pre-reactive complex. This deeply submerged transition state facilitates rapid reaction and leads to the formation of the functionalized hydroperoxide species, HPMAF. The transition state involves the concerted transfer of a H-atom from formic acid to the terminal oxygen of MACR-oxide, while a bond is formed between the carbonyl O-atom of formic acid and the central C-atom of MACR-oxide as shown in Scheme 2. Finally, we consider spectator catalysis of *syn*-MACR-oxide to dioxole products (Figure S6) but find a significant transition state barrier (3.2 kcal mol^−1^) indicating this pathway is not competitive with the more favorable 1,4-addition mechanism (see Supplementary Materials Section S2). Additional details regarding the electronic structure calculations, including analogous reaction pathways characterized for the *syn* conformers of MACR-oxide, and tabulated electronic energies and corrections, are provided in Section S2 of the Supplementary Materials (Table S1 and Figures S4–S6).

**Figure 4:**
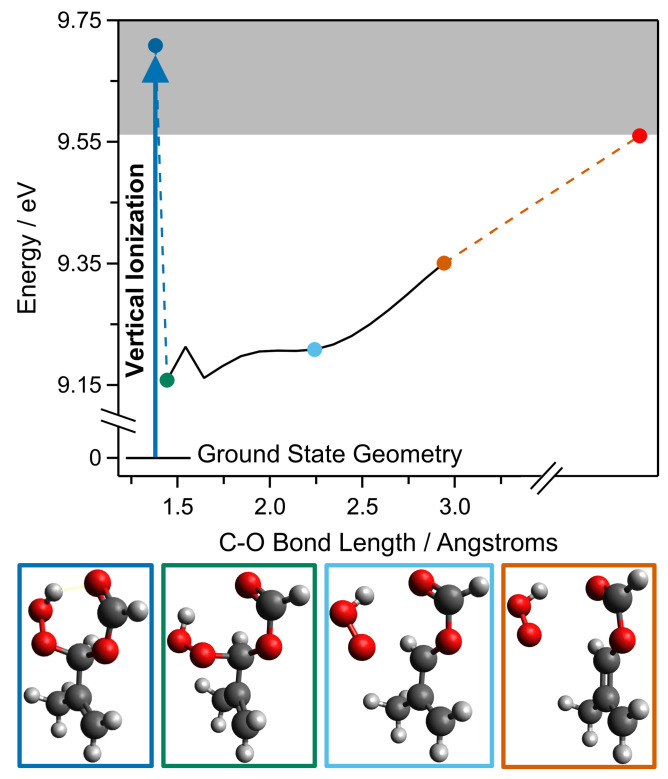
Constrained optimization (ωB97XD/6–31 + G*) of the C–O bond length associated with HO_2_ dissociation from the HPMAF ion. Significant points included are: Franck–Condon excitation from the equilibrium geometry of the ground state (dark blue), the adiabatic minimum of the ion state (green), an intermediate point along the HO_2_ dissociation coordinate (light blue), separated products in the relaxed optimization (orange), and (red) asymptotic energy for the HO_2_-loss fragment ion. The grey shaded region illustrates that the HO_2_ + ion co-fragment asymptote is below the calculated vertical ionization energy, suggesting the photoionization process will result in dissociative ionization.

The observation of a fragment ion associated with HO_2_-loss (*m*/*z* 99, Scheme 1) is characteristic of the photoionization of functionalized hydroperoxide species [12,33,47]. Theoretical calculations were performed to complement the experimental observation of the HO_2_-loss fragment ion (*m*/*z* 99) and absence of signal at the parent mass of HPMAF (*m*/*z* 132) upon photoionization. A schematic plot illustrating the minimum energy path for dissociation of the HPMAF ion to the HO_2_ radical and ion co-fragment along the C–O bond coordinate is shown in Figure 4 at the ωB97XD/6–31 + G* level of theory. Figure 4 illustrates vertical excitation from the optimized ground state geometry of the most stable conformer of HPMAF to the ion state (dark blue). The relaxation energy associated with the Franck–Condon geometry to the minimum of the ion state is evaluated to obtain the adiabatic ionization energy (AIE, blue dashed line to green point). The minimum energy path to HO_2_-loss is obtained through constrained optimizations (stepping the C–O bond length while allowing all other degrees of freedom to optimize, ωB97XD/6–31 + G*) of the equilibrium geometry of the ion (green), through an intermediate configuration (light blue), and finally to full separation of HO_2_ and fragment ion co-products (orange dashed line). The asymptotic energy associated with the fully separated products is obtained from separate calculations of the HO_2_ radical and ion co-products (red point). The constrained optimizations indicate there is no transition state barrier to dissociation of the HPMAF ion. In addition, the asymptotic energy for HO_2_-loss is substantially below the vertical ionization energy (VIE) for HPMAF. Thus, we expect to observe the HO_2_-loss fragment ion at VUV photon energies greater than the calculated asymptotic energy (grey shaded region).

Higher level calculations of the critical points along the fragmentation pathway are evaluated at the CCSD(T)-F12/cc-pVTZ-F12//B2PLYP-D3/cc-pVTZ (CCSD(T)/TZF) level and provided in Table 1.

The higher level CCSD(T)/TZF calculations reveal that C-O bond fission from the adiabatic minimum requires only 0.16 eV (3.7 kcal mol^−1^). This indicates the HO_2_ group is weakly bound in the HPMAF ion such that dissociation is facile at VUV energies greater than 9.89 eV. In addition, there are significant differences in the optimized geometries of the ground and ion states (Figure 4) suggesting Franck–Condon overlap may be weak near the threshold for ionization of HPMAF. This poor overlap is evidenced by a significant difference in the calculated AIE and VIE of HPMAF (0.45 eV). The combination of small Franck–Condon overlap near the threshold for ionization and the small amount of energy required for HO_2_-loss from the HPMAF ion suggests the HPMAF parent signal at *m*/*z* 132 may be weak or missing, consistent with the experimental result.

**Figure 5:**
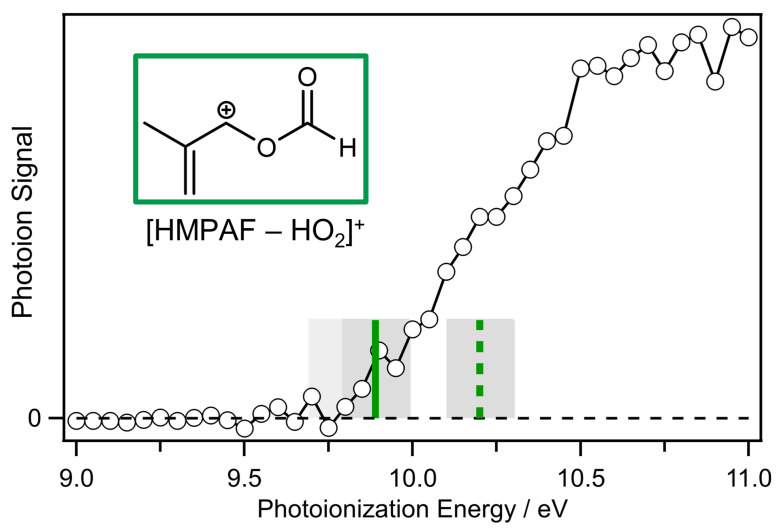
Photoionization spectrum of *m*/*z* 99 obtained upon introduction of formic acid (2.6 × 10^13^ cm^−3^) and integration over the full kinetic time window (0–60 ms) with calculated appearance energy of the HMPAF–HO_2_ fragment ion (green solid line) and vertical ionization energy of HPMAF (green dashed line). The grey shaded region represents uncertainty associated with the calculated ionization energies (±0.1 eV). The light grey shaded region represents the appearance energy of higher energy conformers of HPMAF.

Further evidence for the formation of HPMAF is obtained by comparing the photoionization spectrum of the *m*/*z* 99 product with the calculated asymptotic energy of the HO_2_-loss fragment ion. The photoionization spectrum of the *m*/*z* 99 product integrated over the full kinetic time window (0–60 ms) for each VUV photon energy (9.0–11.0 eV, 50 meV steps) is shown in Figure 5. The asymptotic energies computed for HO_2_-loss (9.89 eV) and the VIE of HPMAF (10.18 eV) are shown by the green solid and dashed lines, respectively. Uncertainty in the calculated ionization energies is represented by the grey shaded region (± 0.1 eV). Higher energy conformers of HPMAF are likely populated under the experimental conditions (298 K = 25 °C, 10 torr = 13.3 hPa), and would have a lower appearance energy than the most stable conformer by up to 0.2 eV (light grey shaded region). There is good agreement between the observed and calculated appearance energy for HO_2_-loss from HPMAF. Thus, the HO_2_-loss mass channel (*m*/*z* 99) provides direct evidence for the formation of HPMAF from the reaction of MACR-oxide with formic acid. The photoionization spectrum of the *m*/*z* 87 mass channel integrated over the full kinetic time window (0–60 ms) for each VUV photon energy (9.0–11.0 eV, 50 meV steps) is shown in Figure S3, which reveals low and high energy components suggestive of multiple species contributing to the photoionization signal. The appearance energy of the higher energy component agrees well with the asymptotic energy computed for the HCO_2_-loss fragment ion shown in Scheme 2. The origin of the lower energy component is discussed in Section S1 of the Supplementary Materials.

**Figure 6:**
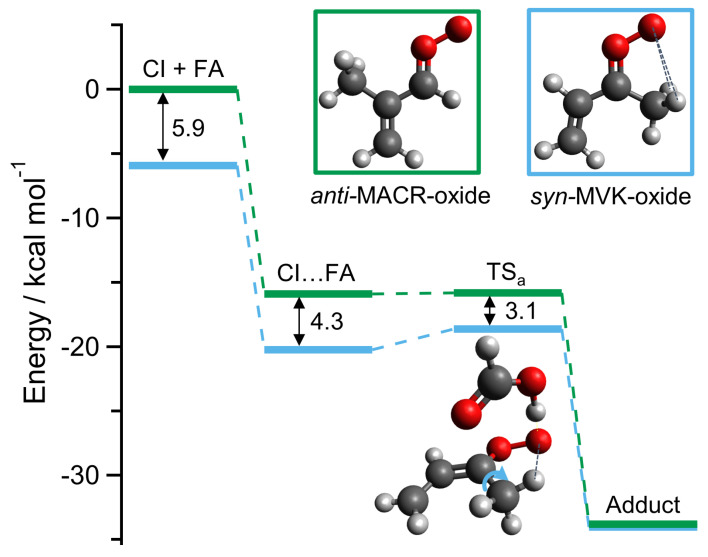
Reaction coordinate comparing the absolute energies for the reaction of formic acid (FA) with *anti*-*trans*-MACR-oxide (green) and *syn*-*trans*-MVK-oxide (teal) Criegee intermediates (CIs). Energies for the reaction of *syn*-*trans*-MVK-oxide are adapted from Ref. [33] with permission from the PCCP Owner Societies. All energies are reported in kcal mol^−1^ at the CCSD(T)-F12/TZF level of theory.

Analogous electronic structure calculations were previously carried out to study the reaction of MVK-oxide with formic acid [12,33]. MVK-oxide is the other four-carbon unsaturated Criegee intermediate that can be formed from isoprene ozonolysis. Since MVK-oxide and MACR-oxide are isomers of each other, a direct comparison of the reaction energetics is especially meaningful. A comparison of the absolute energetics for the reaction of *anti*-*trans*-MACR-oxide and *syn*-*trans*-MVK-oxide with formic acid is shown in Figure 6. *syn-trans*-MVK-oxide is more stable than *anti-trans*-MACR-oxide by ca. 5.9 kcal mol^−1^. The stabilization of *syn*-*trans*-MVK-oxide is in part due to the presence of two hydrogen bonding interactions between the terminal O-atom of MVK-oxide with the adjacent H-atoms of the methyl group (illustrated in Figure 6 by grey dashed lines), as observed for the *syn* conformer of the methyl-substituted Criegee intermediate, CH_3_CHOO [48,49]. A similar stabilizing interaction is not available for *anti*-MACR-oxide because the methyl group is adjacent to the vinyl substituent and pointing away from the carbonyl oxide moiety. The difference in energy decreases to ca. 4.3 kcal mol^−1^ upon formation of the pre-reactive complex between the Criegee intermediates and formic acid, likely due to a stronger dipole-dipole interaction between the O-atom of the C=O of formic acid with the central C-atom of the carbonyl oxide group of *anti*-MACR-oxide. The stronger dipole-dipole interaction is manifested in the smaller intermolecular distance in *anti*-MACR-oxide…FA (2.485 Å) compared to *syn*-MVK-oxide…FA (2.891 Å). The larger intermolecular distance in the *syn*-MVK-oxide pre-reactive complex is likely due to steric hindrance of the methyl group adjacent to the terminal oxygen. The transition state barrier for the 1,4-addition of formic acid to MVK-oxide is slightly larger (1.6 kcal mol^−1^) than that for MACR-oxide, which is nearly barrierless (0.1 kcal mol^−1^), resulting in an energy difference between the transition states of ca. 3.1 kcal mol^−1^. At the transition state, the distance between the O-atom of formic acid and C-atom of the Criegee intermediate are similar, 2.294 Å and 2.263 Å, for the reactions involving MACR-oxide and MVK-oxide, respectively. The slightly higher barrier for the 1,4-addition of formic acid to MVK-oxide can be explained by the loss of one hydrogen bonding interaction at the transition state due to rotation of its methyl group (Figure 6, teal arrow). The energies of the two systems are nearly identical upon formation of the functionalized hydroperoxide product where the extended conjugation of both systems and the hydrogen bonding interactions present for MVK-oxide are lost.

As a result of the highly submerged reaction pathway, the energetic differences that arise from steric hindrance and disruption of the hydrogen bonding interactions present for MVK-oxide likely do not result in a substantial difference in the rate coefficient for the reaction of formic acid with MVK-oxide (3.0 × 10^−10^ cm^3^ s^−1^) [12] compared to that with MACR-oxide. However, we anticipate that these interactions may play a more significant role in reactions in which the energy of the transition state is similar to that of the reactants, leading to enhanced MACR-oxide bimolecular kinetics compared with MVK-oxide. For example, the conformational form of the methyl-substituted Criegee intermediate with the terminal O-atom oriented away from the methyl group (*anti*-CH_3_CHOO) has been shown to have dramatically enhanced reactivity (ca. 10^3^–10^5^) with reactants, such as water vapor and alcohols, compared to *syn*-CH_3_CHOO [38,42,43,50,51,52,53]. The enhanced reactivity of *anti*-CH_3_CHOO arises from its ability to have more favorable dipole-dipole interactions with reactants than for *syn*-CH_3_CHOO. In addition, there is an energetic consequence for disrupting the hydrogen bonding interaction between the terminal O-atom and methyl substituent for *syn*-CH_3_CHOO that gives rise to a larger transition state barrier for reaction. Thus, we anticipate that the enhanced reactivity of *anti*-MACR-oxide with SO_2_ and water vapor [12,34] may originate from the ability of MACR-oxide to have stronger dipole-dipole interactions with reactants as well as the absence of hydrogen bonding interactions that are present for *syn*-MVK-oxide.

Previous studies of smaller Criegee intermediates have demonstrated that unimolecular decay or reaction with water dimer are important tropospheric sinks [50,54,55,56,57], the latter due to the much higher concentration of water vapor in the troposphere compared with other potential bimolecular reaction partners (e.g., NO_2_, SO_2_, organic acids). By contrast, *anti*-MACR-oxide is predicted to have a relatively long atmospheric lifetime compared to other Criegee intermediates due to its slow predicted unimolecular decay rate (ca. 10 s^−1^, 298 K) [38] and small effective rate coefficient with water monomer and dimer ((9 ± 5) × 10^−17^ cm^3^ s^−1^); and thus other bimolecular reactions may be important [34]. The large rate coefficient measured for the reaction of *anti*-MACR-oxide with SO_2_ [34], and the large anticipated rate coefficient for its reaction with formic acid due to the highly submerged reaction pathway (~ 10^−10^ cm^3^ s^−1^) [12] mapped out in this work indicates these reactions can compete for *anti*-MACR-oxide removal in environments with abundant SO_2_ and formic acid. Global modeling indicates the reaction of MVK-oxide with SO_2_ contributes to sulfuric acid production, ultimately enhancing the formation of sulfate aerosols [12]. In addition, the reaction of MVK-oxide with formic acid leads to ca. 20% reduction in modeled formic acid globally. We anticipate that the reaction of MACR-oxide with formic acid will play a similar role to MVK-oxide in the removal of formic acid in areas with large isoprene emissions such as the Amazon [12], and generate functionalized hydroperoxide species that may be precursors to secondary organic aerosols [16].

## 3. Materials and Methods

### 3.1. Experimental Methods

Products from the reaction of MACR-oxide with formic acid are investigated using the Sandia Multiplexed Photoionization Mass Spectrometer (MPIMS) apparatus interfaced with the tunable vacuum ultraviolet (VUV) radiation of the Chemical Dynamics Beamline (9.0.2) of the Advanced Light Source (Lawrence Berkeley National Laboratory, Berkeley, CA, USA) [58]. MACR-oxide is generated by photolysis of the (*E*)-1,3-diiodo-2-methylprop-1-ene precursor and subsequent reaction of the iodoalkenyl radical products with O_2_ as described previously [27,31]. The precursor ((~2–3) × 10^13^ cm^−3^) is entrained in a He flow using a pressure and temperature controlled glass bubbler (100 torr = 133.3 hPa, 298 K = 25 °C) and mixed with O_2_ (~6.4 × 10^16^ cm^−3^) and formic acid in a He bath gas using calibrated mass flow controllers. The gas mixture is delivered to a halocarbon wax-coated quartz reactor tube (298 K = 25 °C, 10 torr = 13.3 hPa), which is photolyzed along its length with the 248 nm output of a KrF excimer laser (40 mJ cm^−2^ pulse^−1^). The laser energy is selected to reduce the concentration of MACR-oxide (max. ~1 × 10^12^ cm^−3^) in order to minimize secondary chemistry. Reactants, intermediates, and products are continuously sampled from an orifice (~650 µm) in the reactor sidewall into vacuum. The resultant free jet expansion is skimmed and intercepted orthogonally by tunable VUV radiation. Ions generated via absorption of the VUV radiation are pulse extracted by ion optics and detected using orthogonal acceleration time-of-flight mass spectrometry. Products resulting from the reaction of MACR-oxide with formic acid are investigated with fixed ionization energy (10.5 eV) and photoionization scans (9.0–11.0 eV, 50 meV steps).

### 3.2. Theoretical Methods

Complementary electronic structure calculations are carried out to characterize the energies of the MACR-oxide conformers, the barriers to *cis*/*trans* isomerization, and the stationary points for the reaction of the MACR-oxide conformers with formic acid. The stationary points for the reaction of the four conformers of MACR-oxide with formic acid are determined at the CCSD(T)-F12/cc-pVTZ-F12//B2PLYP-D3/cc-pVTZ (CCSD(T)/TZF) level of theory including zero-point energy (ZPE) corrections. An estimation of the CCSDT(Q) correction for higher order excitations in the coupled cluster expansion is also incorporated to account for multireference effects. The estimate is based on CCSDT(Q) corrections calculated for the isomerization of MVK-oxide [28] and the chemical reaction of CH_2_OO with SO_2_ [12].

Additional electronic structure calculations are carried out to investigate the minimum energy path to fragmentation of the HPMAF ion (HO_2_-loss, CH_3_C(=CH_2_)C^+^HOOH) following VUV photoionization. The geometry of the HPMAF ion is optimized and the minimum energy path to HO_2_-loss is mapped through constrained optimizations of the C–O bond length at the ωB97XD/6–31 + G* level. Higher level calculations are carried out to obtain the vertical and adiabatic ionization energies of HPMAF, and appearance energy of the HO_2_-loss fragment ion at the CCSD(T)/TZF level. The appearance energy of the HO_2_-loss fragment ion is obtained by summing the energies of separate optimizations of the HO_2_ and fragment ion. ZPE corrections are included for the adiabatic ionization energy and appearance energy of the HO_2_-loss fragment ion. The ωB97XD and B2PLYP-D3 are performed using Gaussian16 [59], whereas the CCSD(T)-F12 calculations are done using Molpro 2015 [60].

## 4. Conclusions

In this study, we perform high level ab initio calculations that show there is a highly submerged pathway for the reaction of MACR-oxide with formic acid via a 1,4-addition mechanism that generates a functionalized hydroperoxide species, HPMAF. We identify HPMAF from the reaction of MACR-oxide with formic acid with photoionization (MPIMS) detection. Specifically, dissociative photoionization of HPMAF is observed by the identification of fragment ions associated with HO_2_-loss (*m*/*z* 99) and HCO_2_-loss (*m*/*z* 87) processes characteristic of functionalized hydroperoxides. Rapid appearance of photoionization signal at *m*/*z* 99 and 87 is consistent with the highly submerged pathway for reaction. The experimental fragment ion appearance energies agree with calculations of their appearance energies, providing further support for the formation of HPMAF in the reaction of MACR-oxide with formic acid. Additional calculations mapping out the minimum energy path for dissociation of the HPMAF ion to the HO_2_-loss fragment ion indicates the asymptote for dissociation is below the vertical ionization energy associated with Franck–Condon excitation from the equilibrium geometry of the ground state to the ion, suggesting that the ionization process will directly produce the HO_2_-loss fragment ion, as is observed experimentally.

In addition, we compare the energetics for the reaction of formic acid with MVK-oxide, the other four-carbon resonance stabilized Criegee intermediate formed from isoprene ozonolysis, with that for MACR-oxide. The hydrogen bonding interaction present in the *syn*-MVK-oxide system between the terminal O-atom of the carbonyl oxide group and adjacent H-atoms of the methyl group as well as steric hindrance from the methyl group appear to influence the reaction profile. Disruption of one of the hydrogen bonds at the transition state due to rotation of the methyl group leads to an increased barrier for reaction of *syn*-MVK-oxide with formic acid than that for *anti*-MACR-oxide, which lacks a methyl group adjacent to the terminal O-atom. We anticipate that this difference will result in minor perturbations to the rate coefficient for reaction of *anti*-MACR-oxide with formic acid compared to that with *syn*-MVK-oxide. More significant effects may be observed for the reactions of four-carbon Criegee intermediates with water vapor or SO_2_, which typically have substantially larger transition state barriers. Due to the relatively long lifetime predicted for *anti*-MACR-oxide with respect to unimolecular decay and reaction with water vapor, we anticipate that the reaction of *anti*-MACR-oxide with formic acid will contribute to the removal of formic acid in regions with high isoprene emissions and form highly oxidized hydroperoxide species that may play a role in secondary organic aerosol formation.

## Figures and Tables

**Table 1:** Vertical ionization energy (VIE) and zero-point energy corrected adiabatic ionization energy (AIE) of HPMAF, and asymptotic energies for the anticipated fragment ions calculated at three levels of theory. All energies are reported relative to the most stable conformational form of HPMAF.

	ωB97XD 6–31 + G*	B2PLYP-D3 cc-pVTZ	CCSD(T)-F12 TZF
VIE (HPMAF)	9.71	9.94	10.18
AIE (HPMAF)	9.16	9.32	9.73
CH_3_C(=CH_2_)C^+^HOOH (HO_2_-loss)	9.58	9.56	9.89
CH_3_C(=CH_2_)C^+^HOC(O)H (HCO_2_-loss)	10.17	10.05	10.53

## Data Availability

All data is available in the main text, in the supplementary materials, or on reasonable request.

## Supplementary Materials

The following are available online, additional experimental details, computed stationary point geometries, and energy corrections. Section S1. HCO_2_-loss fragment ion, Section S2. Theoretical reaction pathways, Section S3. Stationary point geometries, Table S1. Stationary point energies and corrections, Figure S1 Temporal profile of *m*/*z* 87 as a function of formic acid concentration, Figure S2: Comparison of *m*/*z* 87 and 99 integrated signals as a function of formic acid concentration, Figure S3: Photoionization spectrum of *m*/*z* 87 with and without formic acid added, Figure S4. Reaction coordinate for the 1,4-addition of *syn-cis*-MACR-oxide with formic acid, Figure S5. Reaction coordinate for the 1,4-addition of *syn-trans*-MACR-oxide with formic acid, Figure S6. Reaction coordinate for the spectator catalysis of *syn-cis*-MACR-oxide to dioxole.

## Sample Availability

Samples of the compounds are not available from the authors.
