# Supplementary material for: Functionalized Hydroperoxide Formation from the Reaction of Methacrolein-Oxide, an Isoprene-Derived Criegee Intermediate, with Formic Acid: Experiment and Theory
Source: Molecules. 2021 May 20;26(10):3058. doi: 10.3390/molecules26103058 (PMC8161369; doi:10.3390/molecules26103058)
Supplement: Supplementary file 1 [file molecules-26-03058-s001.zip › molecules-1213652-supplementary.pdf]

Supplementary Materials

Functionalized hydroperoxide formation from the reaction of methacrolein-oxide, an isoprene-derived Criegee intermediate, with formic acid: Experiment and theory

Michael F. Vansco[1,2], Kristen Zuraski[3], Frank A. F. Winiberg[4,5], Kendrew Au[6] , Nisalak Trongsiriwat[1], Patrick J. Walsh[1], David L. Osborn[6,7], Carl J. Percival[4], Stephen J. Klippenstein[2], Craig A. Taatjes[6*], Marsha I. Lester[1*], and Rebecca L. Caravan[2,3,*]

[1]Department of Chemistry, University of Pennsylvania, Philadelphia, PA 19104-6323, USA.
[2]Chemical Sciences and Engineering Division, Argonne National Laboratory, Lemont, IL 60439, USA.
[3]NASA Postdoctoral Program Fellow, NASA Jet Propulsion Laboratory, California Institute of Technology, 4800 Oak Grove Drive, Pasadena, CA 91109, USA.
[4]NASA Jet Propulsion Laboratory, California Institute of Technology, 4800 Oak Grove Drive, Pasadena, CA 91109, USA.
[5]Division of Chemistry and Chemical Engineering, California Institute of Technology, Pasadena, CA 91125, USA.
[6]Combustion Research Facility, Mailstop 9055, Sandia National Laboratories, Livermore, CA 94551, USA.
[7]Department of Chemical Engineering, University of California, Davis, CA 95616, USA
Correspondence: cataatj@sandia.gov (C.A.T), milester@sas.upenn.edu (M.I.L.), and caravarl@anl.gov (R.L.C.)

Table of Contents:

Section S1. HCO_2-loss fragment ion……………………………………………………………...S3

Section S2. Theoretical reaction pathways……………………………………………………..S6

Section S3. Stationary point geometries………………………………………………………S14

Table S1. Stationary point energies and corrections……………………………………………S8

Figure S1 Temporal profile of m/z 87 as a function of formic acid concentration……………….S3

Figure S2: Comparison of m/z 87 and 99 integrated signals as a function of formic acid concentration...S4

Figure S3: Photoionization spectrum of m/z 87 with and without formic acid added……………S5

Figure S4. Reaction coordinate for the 1,4-addition of *syn-cis*-MACR-oxide with formic acid………..S11

Figure S5. Reaction coordinate for the 1,4-addition of *syn-trans*-MACR-oxide with formic acid……...S12

Figure S6. Reaction coordinate for the spectator catalysis of *syn-cis*-MACR-oxide to dioxole………...S13

Section S1. HCO_2-loss fragment ion

Functionalized hydroperoxides formed from the reaction of Criegee intermediates with formic acid have been shown to undergo dissociative ionization to HO_2-loss and HCO_2-loss fragment ions [1]. Scheme 2 (main text) shows the fragment ions that are predicted to be generated from dissociative ionization of 1-hydroperoxy-2-methylallyl formate (HPMAF), the functionalized hydroperoxide predicted to be formed from the reaction of MACR-oxide with formic acid. The chemical composition of the fragment ions, $C_5H_7O_2$ (99.045) and $C_4H_7O_2$ (87.045), are consistent with the exact masses of the product features observed in the experiment (99.045 ± 0.002 and 87.043 ± 0.003, respectively). The time profile of m/z 99 (Figure S1) obtained using 10.5 eV VUV photon energy shows a weak signal is present at m/z 87 in the absence of formic acid (light grey line).

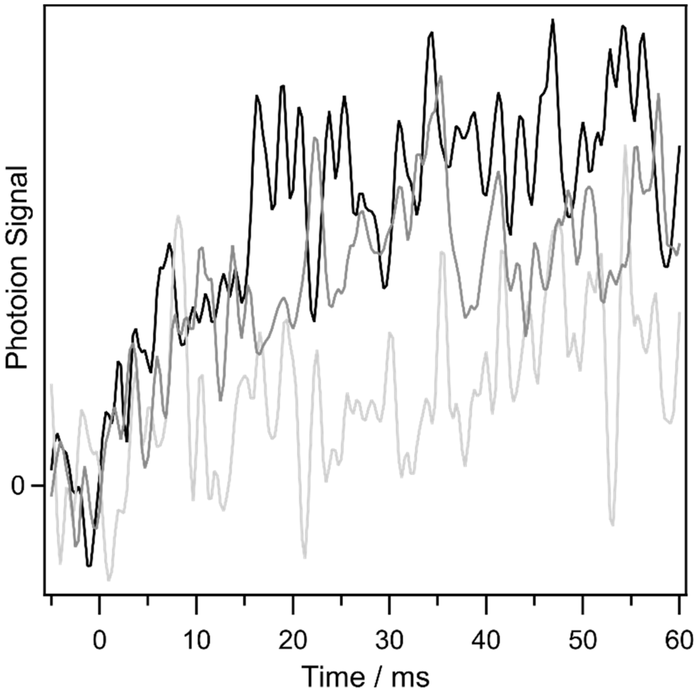

Figure S1. Temporal profile of m/z 87 observed from the reaction of MACR-oxide with formic acid (light grey line [formic acid] = 0 cm^{-3}, dark grey line [formic acid] = 6.6×10^{12} cm^{-3}, black line [formic acid] = 5.7×10^{13} cm^{-3}) at a photoionization energy of 10.5 eV. A three-point smooth is applied to the data to guide the eye. A weak signal is present at m/z 87 in the absence of formic acid. The amplitude of the m/z 87 signal increases in the presence of formic acid and reveals the rapid formation of a stable product

The signal at m/z 87 increases in amplitude with the addition of formic acid and exhibits a similar time profile to that of m/z 99 providing evidence that the formic acid dependent signals originate from the same source. Figure S2 shows the integrated signal of m/z 99 (left axis) and 87 (right axis) at a VUV photon energy of 10.5 eV as a function of formic acid concentration.

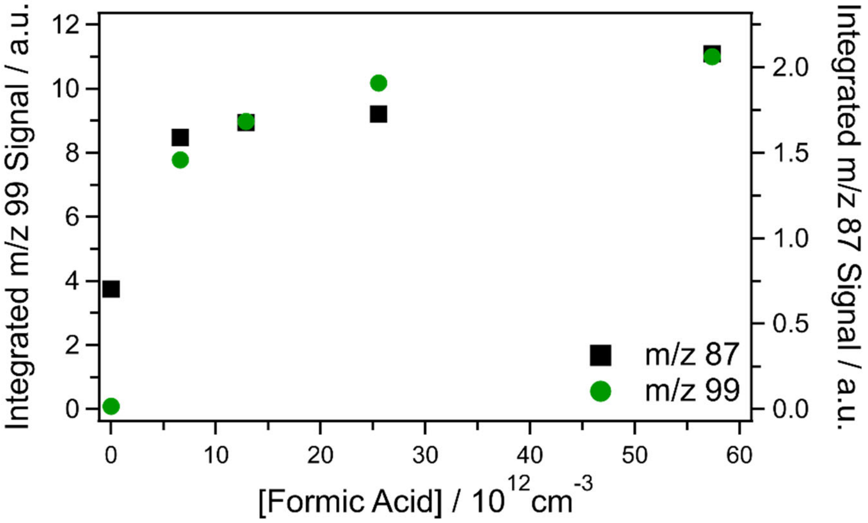

Figure S2. Integrated signal of m/z 99 (left, green circles) and m/z 87 (right, black squares) obtained using a photon energy of 10.5 eV as a function of formic acid concentration.

The signals at m/z 99 and 87 show a similar increase with increasing formic acid concentration, indicating the formic acid dependent signal originates from the same source. Assuming the reaction of MACR-oxide with formic acid has a similar rate coefficient as the reaction of MVK-oxide with formic acid (3×10^{-10} cm^3 s^{-1}), a slow predicted unimolecular decay rate (~10 s^{-1}) [2] and a wall loss of ~400 s^{-1}, the majority of the initial thermalized MACR-oxide population (~80%) should react with formic acid even at the lowest formic acid concentration used in the experiment (6.6×10^{12} cm^{-3}). This prediction is consistent with the small increase in amplitude of the fragment ion signals at formic acid concentrations >6.6×10^{12} cm^{-3} providing evidence that the formic acid dependent signal on m/z 87 originates from reaction of MACR-oxide with formic acid.

Detection of the HCO_2-loss fragment ion from photoionization of HPMAF is further evidenced by comparing the photoionization spectrum of m/z 87 to calculations of its appearance energy at the

CCSD(T)-F12/cc-pVTZ-F12//B2PLYP-D3/cc-pVTZ level of theory. Figure S3 shows the photoionization spectrum of m/z 87 in the presence (9.0-11.0 eV, 50 meV steps, open circles) and absence (9.0-10.7 eV, 50 meV steps, red line) of formic acid integrated over the full kinetic time window (0-60 ms).

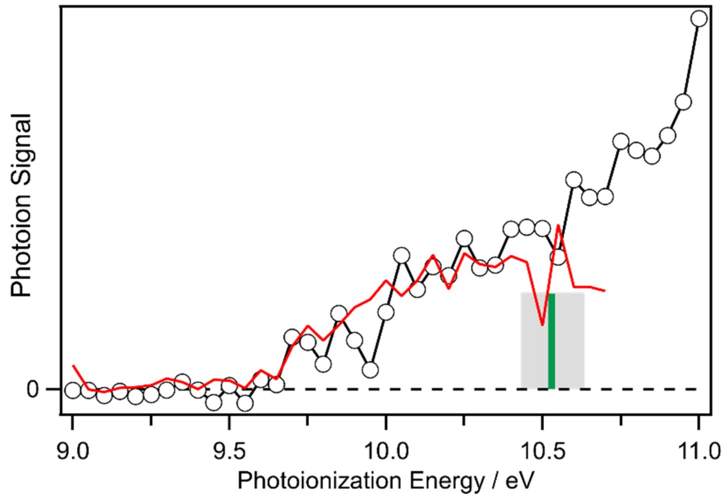

Figure S3. Photoionization spectrum of m/z 87 obtained in the presence (open circles, [formic acid] = 2.6×10^{13} cm^{-3}) and absence (red line, [formic acid] = 0 cm^{-3}) of formic acid and integrated over the full kinetic time window (0-60 ms). Comparison to the photoionization spectrum obtained in the absence of formic acid reveals two components contribute to the m/z 87 photoionization signal. The calculated appearance energy of the HPMAF–HCO_2 fragment ion (green solid line, 10.53 eV) agrees well with the higher energy component. The grey shaded region represents uncertainty associated with the calculated ionization energies (±0.1 eV).

The photoionization spectrum of m/z 87 obtained in the presence of formic acid reveals low and high energy components. The low energy component is consistent with the photoionization spectrum of m/z 87 in the absence of formic acid (red trace). The persistence of the lower energy component after addition of formic acid, an effective Criegee intermediate scavenger, indicates its origin is likely not from reactions involving MACR-oxide. The higher energy component is consistent with calculations of the appearance energy of the HCO_2-loss fragment ion (10.53 eV), confirming the formic acid dependent signal originates from its reaction with MACR-oxide. The relatively small signal on m/z 87 signal compared to m/z 99 obtained at 10.5 eV as a function of formic acid concentration is consistent with the VUV photon energy being near the threshold for dissociative ionization of HPMAF to HCO_2 and fragment ion co-product.

Section S2: Theoretical reaction pathways

Overview

The reaction of MACR-oxide with formic acid is investigated with electronic structure calculations for all four conformational forms of MACR-oxide. The results for HPMAF formation from the reaction of *anti-trans*-MACR-oxide, the most stable conformer of MACR-oxide, with formic acid is shown in the main text. Here, we present results for the reaction of *anti-cis*-MACR-oxide, *syn-cis*-MACR-oxide, and *syn-trans*-MACR-oxide with formic acid. In addition, we present the results regarding the spectator catalysis of *syn-cis*-MACR-oxide by formic acid to dioxole.

Stationary Point Energies

The stationary point energies for the various reaction channels in the reaction of all four conformers of MACR-oxide with formic acid were determined at the CCSD(T)-F12/cc-pVTZ-F12//B2PLYP-D3/cc-pVTZ level of theory with an estimated CCSDT(Q) correction based on previous calculations for the isomerization of MVK-oxide and reaction of CH_2OO with SO_2 [1, 3], to account for multireference effects present for Criegee intermediates. These effects arise from resonances between zwitterionic and singlet diradical electronic configurations of Criegee intermediates. Calculations at the CCSDT(Q) level largely capture these multireference effects, but such calculations are impractical for the many heavy atom system being investigated. Specifically, the MACR-oxide pre-reactive complexes (PRCs), transition states for chemical conversions from the PRCs, and products are estimated to be raised by 0.4, 0.8, and 1.5 kcal mol^{-1} relative to the reactants, respectively. We estimate the uncertainties in these corrections to be ~0.2 kcal mol^{-1} for the PRCs and transition states, and ~0.4 kcal mol^{-1} for the products. Zero-point energy (ZPE) corrections are evaluated at the B2PLYP-D3/cc-pVTZ level. Overall, we expect 2σ uncertainties in our predicted energies of ~0.6 kcal mol^{-1}. The resultant energies are reported in Table S1.

The reaction pathway for HPMAF formation from the reaction of *anti-trans*-MACR-oxide, the lowest energy conformer, is illustrated in the main text (Figure 6). The analogous reaction pathways for

HPMAF formation from the reactions of *syn-trans*-MACR-oxide and *syn-cis*-MACR-oxide are illustrated in Figure S4 and S5. The reaction is predicted to proceed via a similar 1,4-addition mechanism via a highly submerged transition state barrier to form HPMAF. Interestingly, the reaction of *anti-cis*-MACR-oxide with formic acid is barrierless at the B2PLYP-D3/cc-pVTZ level of theory. At the ωB97XD/6-31+G* level of theory, a stationary point for the pre-reactive complex and first-order saddle point for the transition state to HPMAF formation is found. However, the zero-point energy of the pre-reactive complex is larger than that of the transition state such that the reaction is effectively barrierless at the ωB97XD/6-31+G* level of theory as well.

Table S1. Stationary point energies (kcal mol^{-1}) for the reaction of *syn/anti-cis/trans* (*s/a-c/t*) MACR-oxide with formic acid (FA).[a] Energies include ZPE corrections and are reported relative to *a-t*-MACR-oxide.

Stationary Point	ωB97XD 6-31+G*	B2PLYP-D3 cc-pVTZ	CCSD(T)-F12 cc-pVTZ-F12	E0[b]	Total[c]	Corr. Total[d]
Reactants						
a-t-MACR-oxide + FA	0	0	0	0	0	0
s-c-MACR-oxide + FA	-0.3	0.5	0.7	0.2	0.9	0.9
s-t-MACR-oxide + FA	1.7	2.2	2.3	0.2	2.5	2.5
a-c-MACR-oxide + FA	2.7	3.7	3.4	-0.2	3.2	3.2
van der Waals						
Reactant Wells						
a-t-MACR-oxide…FA	-20.8	-19.2	-17.2	0.9	-16.3	-15.9
s-c-MACR-oxide…FA	-18.7	-15.9	-13.6	1.3	-12.3	-11.9
s-t-MACR-oxide…FA	-17.9	-15.8	-13.8	1.3	-12.5	-12.1
a-c-MACR-oxide…FA	-19.0	N/A	N/A	N/A	N/A	N/A
Product Wells						
Dioxole…FA	-45.4	-38.7	-39.4	2.6	-36.8	-35.3
Transition States						
cis/trans						
a-t-MACR-oxide = *a-c*-MACR-oxide	9.4	10.2	9.3	-0.5	8.8	9.2
s-t-MACR-oxide = *s-c*-MACR-oxide	9.4	9.6	8.9	-0.3	8.6	9.0
1,4 Addition (TS_a)						
a-t-MACR-oxide…FA = *a-t*-HPMAF	-20.4	-19.1	-16.6	0.0	-16.6	-15.8
s-c-MACR-oxide…FA = *s-c*-HPMAF	-17.3	-15.0	-11.9	0.0	-11.9	-11.1
s-t-MACR-oxide…FA = *s-t*-HPMAF	-17.0	-15.0	-12.3	0.1	-12.2	-11.4
a-c-MACR-oxide…FA = *a-c*-HPMAF	-19.0	N/A	N/A	N/A	N/A	N/A
Dioxole Formation; w and w/o Spectator Catalysis (TS_d, $TS_{d,cat}$)						
a-c-MACR-oxide = Dioxole	12.4	12.5	13.6	-0.1	13.5	14.3
a-c-MACR-oxide…FA = Dioxole…FA	-3.4	-2.0	1.4	1.0	2.4	3.2
Products						
a-t-HPMAF	-43.5	-37.1	-38.5	3.2	-35.3	-33.8
a-t-HPMAF; Geometry 2	-41.9	-36.3	-38.0	2.7	-35.3	-33.8
s-c-HPMAF	-41.2	-34.1	-35.1	3.3	-31.8	-30.3
s-c-HPMAF; Geometry 2	-39.4	-32.7	-33.8	3.2	-30.6	-29.1
s-t-HPMAF	-42.6	-35.9	-37.2	3.4	-33.8	-32.3
s-t-HPMAF; Geometry 2	-39.7	-34.0	-35.5	2.7	-32.8	-31.3
a-c-HPMAF	-43.5	-37.0	-38.4	3.2	-35.2	-33.7
a-c-HPMAF; Geometry 2	-40.9	-35.1	-36.9	2.7	-34.2	-32.7
Dioxole + FA	-33.4	-28.5	-29.7	1.2	-28.5	-27.0

[a] All energies are relative to *anti-trans*-MACR-oxide + FA. The first three columns are electronic energies, the next is the zero-point vibrational energy. The last two columns are the sum of the CCSD(T)-

F12/cc-pVTZ-F12 electronic energy and B2PLYP-D3/cc-pVTZ zero-point without (Total) and with (Corr. Total) the adhoc correction for multireference effects.

[b] Zero-point energies from harmonic B2PLYP-D3/cc-pVTZ calculations.

[c] Total corresponds to the sum of the CCSD(T)-F12 energy and E0.

[d] The total energy corrected by estimated CCSDT(Q) higher level corrections.

Catalytic reaction pathways

Recently, this group reported direct evidence for the formic acid catalyzed isomerization of *syn*-MVK-oxide to a vinyl hydroperoxide species using MPIMS [4]. Performing the reaction with deuterated formic acid enabled a migration of a D atom to yield a partially deuterated vinyl hydroperoxide product, which was identified by its distinct mass and photoionization threshold. An analogous mechanism is not feasible for the reaction of MACR-oxide with formic acid because MACR-oxide lacks an α-H-atom in a methyl (or alkyl) group adjacent to the terminal O-atom. Specific conformational forms of MACR-oxide (*syn*) are predicted to undergo unimolecular decay via a rapid electrocyclic ring closure mechanism to form a dioxole species. Here, we consider the formic acid catalyzed formation of the dioxole species via a spectator catalysis mechanism (Figure S6). However, similar to the analogous reaction pathway for MVK-oxide, we find that the transition state barrier (3.17 kcal mol^{-1}) is too high to compete with the substantially more favorable 1,4-addition pathway.

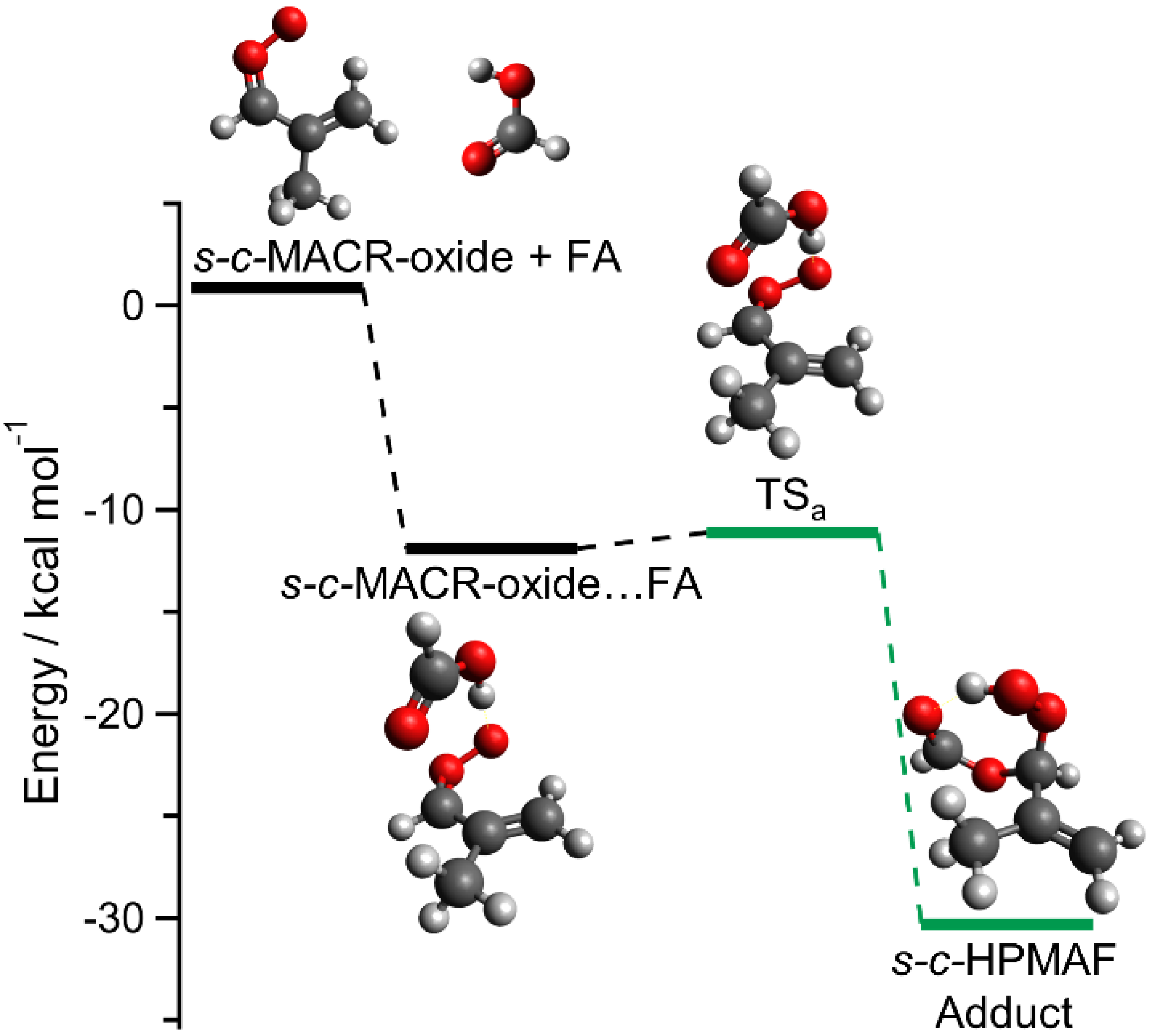

Figure S4. Reaction coordinate showing product formation from the 1,4-addition reaction of *syn-cis*-MACR-oxide with formic acid at the CCSD(T)-F12/cc-pVTX-F12//B2PLYP-D3/cc-pVTZ level of theory including estimated CCSDT(Q) corrections.

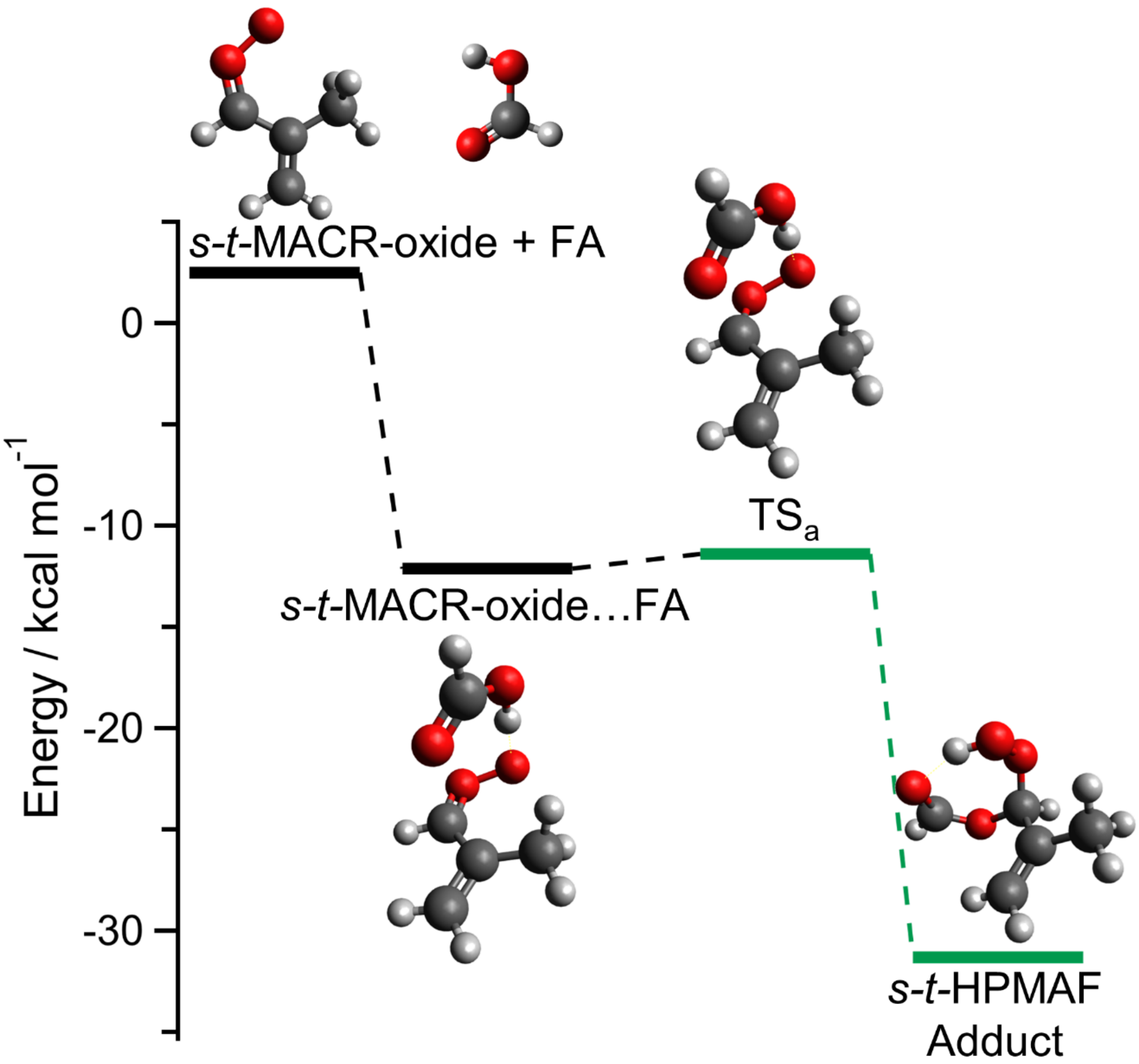

Figure S5. Reaction coordinate showing product formation from the 1,4-addition reaction of *syn-trans*-MACR-oxide with formic acid at the CCSD(T)-F12/cc-pVTX-F12//B2PLYP-D3/cc-pVTZ level of theory including estimated CCSDT(Q) corrections.

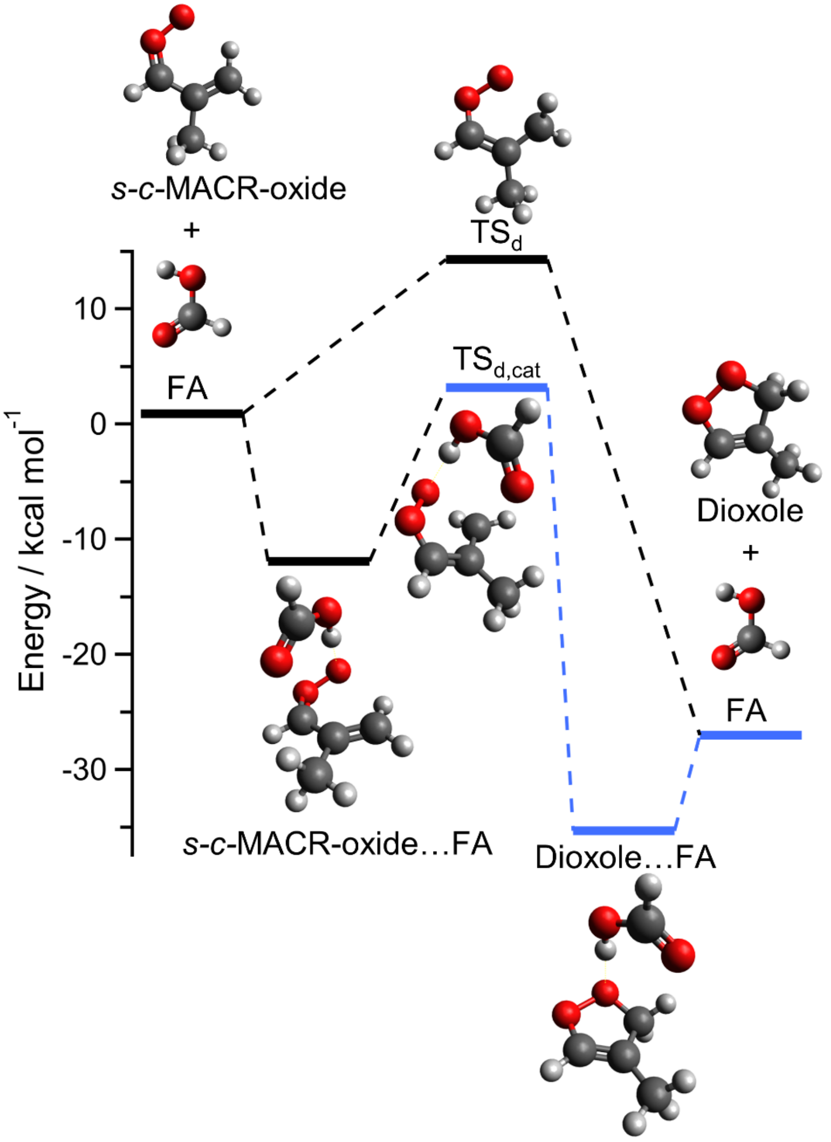

Figure S6. Reaction coordinate showing the spectator catalysis *syn-cis*-MACR-oxide with formic acid to dioxole at the CCSD(T)-F12/cc-pVTX-F12//B2PLYP-D3/cc-pVTZ level of theory including estimated CCSDT(Q) corrections.

Section S3. Stationary point geometries

Structure and Frequencies at the B2PLYP-D3/cc-pVTZ level

Reactants

Formic Acid

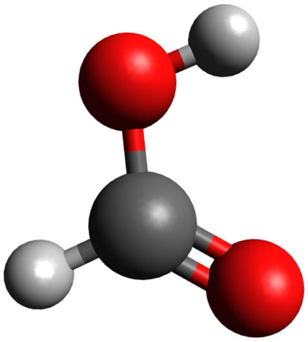

Coordinates
8 -1.0306350000 -0.4423620000 0.0000000000
6 0.0000000000 0.4212380000 0.0000000000
1 -0.6519010000 -1.3342230000 0.0000000000
1 -0.3801630000 1.4461820000 0.0000000000
8 1.1596430000 0.1124390000 0.0000000000

Frequencies

629.737
685.904
1061.85
1130.39
1313.81
1413.54
1812.84
3088.84
3745.69

MACR-oxide

anti-trans-MACR-oxide

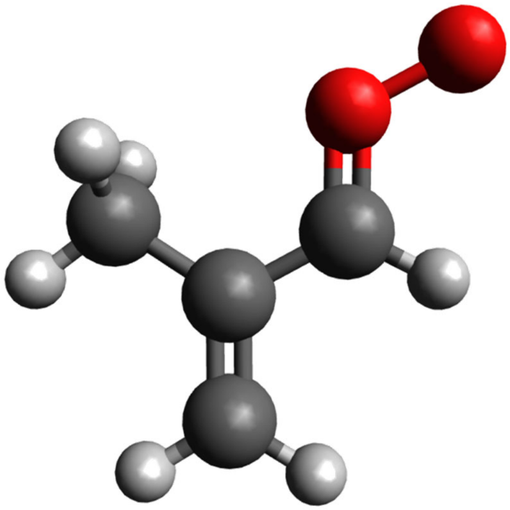

Coordinates
6 2.2150130000 -0.1997910000 0.0000000000
1 2.5967870000 0.8111370000 0.0000000000
1 2.9340580000 -1.0048510000 0.0000000000
6 0.8920210000 -0.4472490000 0.0000000000
6 0.3100810000 -1.8307340000 0.0000000000
1 1.1009980000 -2.5754780000 0.0000000000
1 -0.3196150000 -1.9850150000 0.8751040000
1 -0.3196150000 -1.9850150000 -0.8751040000
6 0.0000000000 0.6736280000 0.0000000000
1 0.3190290000 1.7086680000 0.0000000000
8 -1.2626950000 0.4625590000 0.0000000000
8 -2.0890970000 1.5193700000 0.0000000000

Frequencies

152.383	918.175	1473.1
186.444	940.667	1492.79
188.285	1004.01	1512.67
230.51	1045.05	1655.03
370.822	1060.09	3064.25
479.909	1079.86	3124.54
507.426	1289.9	3154.09
575.991	1370.75	3167.56
698.285	1422.04	3189.08
873.755	1442.39	3260.36

syn-cis-MACR-oxide

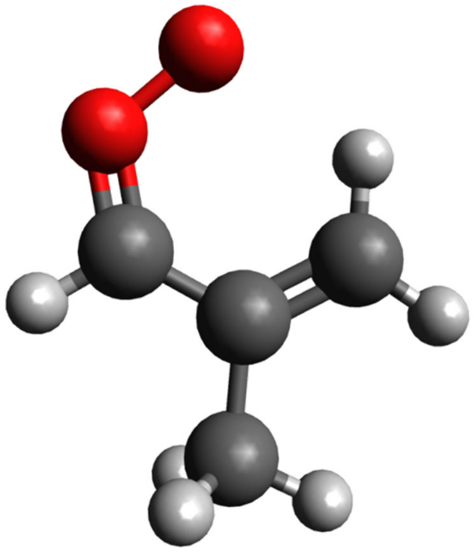

Coordinates
6 -1.3504190000 0.7604020000 0.0000000000
1 -1.8731280000 -0.1777490000 0.0000000000
1 -1.9056560000 1.6888050000 0.0000000000
6 0.0000000000 0.8039380000 0.0000000000
6 0.7571030000 2.1103670000 0.0000000000
1 0.0667310000 2.9493130000 0.0000000000
1 1.3958310000 2.1966750000 0.8790410000
1 1.3958310000 2.1966750000 -0.8790410000
6 0.8326520000 -0.3582720000 0.0000000000
1 1.9102190000 -0.2662740000 0.0000000000
8 0.4946500000 -1.6012620000 0.0000000000
8 -0.7978800000 -1.9594950000 0.0000000000

Frequencies

108.352	913.049	1497.38
130.543	980.079	1499.39
284.771	980.423	1517.28
318.322	1021.5	1629.14
341.31	1050.55	3054
422.588	1081.75	3111.26
541.984	1273.01	3148.08
709.971	1350.37	3166.33
713.074	1419.92	3204.03
861.824	1438.91	3309.97

syn-trans-MACR-oxide

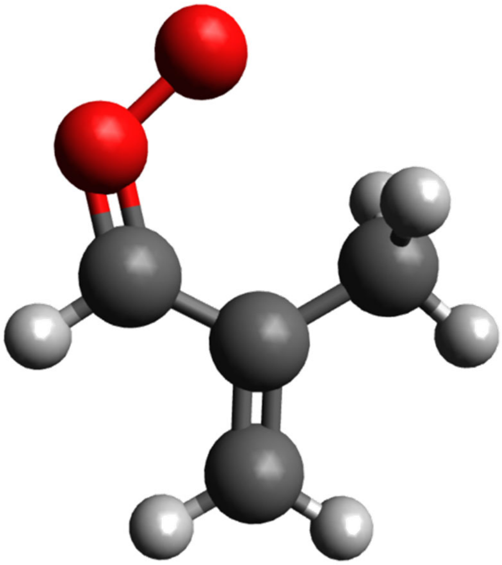

Coordinates
6 -0.5386130000 2.1092540000 0.0000000000
1 -1.6085700000 2.2614290000 0.0000000000
1 0.0873600000 2.9888280000 0.0000000000
6 0.0000000000 0.8728020000 0.0000000000
6 1.4856490000 0.6509730000 0.0000000000
1 1.9953750000 1.6125110000 0.0000000000
1 1.7901250000 0.0737220000 0.8682100000
1 1.7901250000 0.0737220000 -0.8682100000
6 -0.9541260000 -0.2055700000 0.0000000000
1 -2.0138190000 0.0104510000 0.0000000000
8 -0.7475410000 -1.4701750000 0.0000000000
8 0.4977840000 -1.9780020000 0.0000000000

Frequencies

66.2324	858.513	1480.49
213.422	943.713	1481.13
257.608	956.966	1513.44
330.933	1003.97	1642.13
350.161	1068.72	3071.43
426.129	1082.39	3152.58
556.49	1339.05	3154.8
700.598	1358.16	3165.62
729.186	1425.36	3203.68
852.994	1446.01	3257.91

anti-cis-MACR-oxide

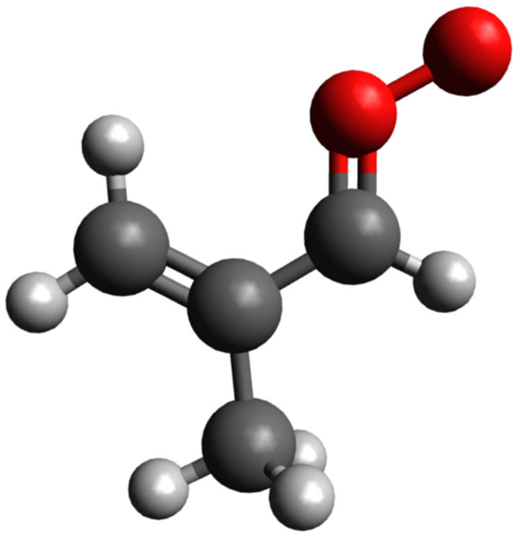

Coordinates
6 -0.0326390000 -1.8490550000 0.0000000000
1 1.0458720000 -1.8928800000 0.0000000000
1 -0.5758180000 -2.7817180000 0.0000000000
6 -0.6881080000 -0.6767650000 0.0000000000
6 -2.1899500000 -0.5821310000 0.0000000000
1 -2.6368090000 -1.5723410000 0.0000000000
1 -2.5479500000 -0.0444610000 0.8783070000
1 -2.5479500000 -0.0444610000 -0.8783070000
6 0.0000000000 0.5929060000 0.0000000000
1 -0.5095460000 1.5490680000 0.0000000000
8 1.2784680000 0.6431150000 0.0000000000
8 1.8760800000 1.8415190000 0.0000000000

Frequencies

92.3566	922.504	1471.8
149.98	946.752	1496.56
198.584	1015.35	1510.56
199.599	1032.84	1662.94
369.885	1076.4	3052.92
466.157	1081.68	3109.33
500.884	1277.93	3150.17
561.715	1318	3175.09
686.124	1426.21	3183.58
912.466	1455.52	3268.36

van der Waals

Pre-reactive Complex (PRC)

anti-trans-MACR-oxide…FA

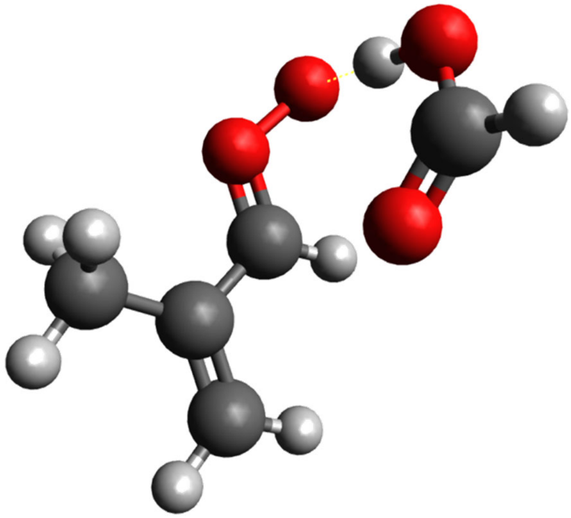

Coordinates
6 -2.3283790000 0.3217350000 1.2494740000
6 -1.8413310000 -0.1361460000 -0.0905310000
6 -0.6023240000 0.3971540000 -0.5977270000
8 -0.0324740000 1.3279350000 0.0362200000
8 1.1873780000 1.7553580000 -0.5317920000
1 -0.1760140000 0.0974410000 -1.5437290000
6 -2.4672900000 -1.0324900000 -0.8667580000
1 -2.0480190000 -1.3409350000 -1.8132910000
1 -3.4004910000 -1.4831130000 -0.5632560000
1 -3.2789040000 -0.1459070000 1.4901900000
1 -1.6026820000 0.0634440000 2.0196650000
1 -2.4501460000 1.4035210000 1.2718930000
8 0.9516870000 -1.4345460000 0.0403200000
6 2.1241930000 -1.2112890000 0.3102620000
8 2.7374370000 -0.0629640000 0.2717390000
1 2.1009280000 0.7026540000 -0.0512040000
1 2.7938840000 -2.0171600000 0.6295140000

Frequencies

47.1659	367.617	1008.54	1458.77	3126.99
53.8529	473.284	1060.54	1492.55	3155.6
77.2663	517.889	1064.41	1504.05	3171.25
158.986	568.005	1082.39	1546.94	3228.22
174.918	713.821	1171.88	1561.42	3264.02
186.865	733.261	1299.04	1681.26	
191.169	875.077	1316.44	1692.9	
242.142	894.257	1395.76	2303.57	
313.528	976.894	1405.1	3056.61	
359.111	991.822	1434.21	3064.79	

syn-cis-MACR-oxide…FA

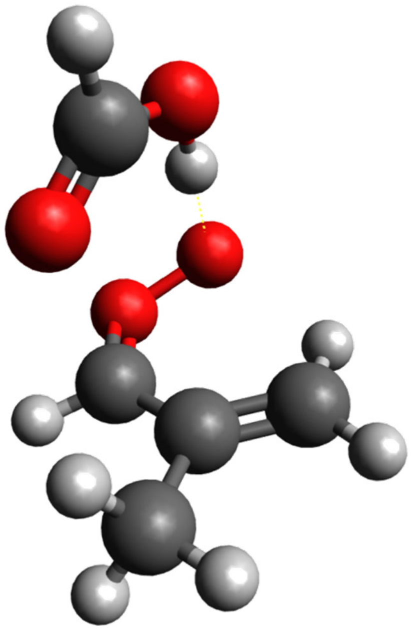

Coordinates
6 -2.3456530000 -1.2434990000 -0.0739910000
6 -1.4965890000 -0.0332550000 0.2101900000
6 -0.8141450000 0.4848270000 -0.9476980000
8 0.0909600000 1.3671320000 -1.0484930000
8 0.6753120000 1.8567190000 0.1145160000
1 -1.1113700000 0.1375490000 -1.9300760000
6 -1.4138500000 0.5335000000 1.4276100000
1 -0.7739760000 1.3788690000 1.6049520000
1 -2.0122330000 0.1367340000 2.2362750000
1 -2.8888640000 -1.5456270000 0.8171390000
1 -3.0696760000 -1.0480300000 -0.8653650000
1 -1.7138090000 -2.0710050000 -0.3942990000
8 0.9587040000 -1.4414060000 -0.5843070000
6 1.9275750000 -1.2420220000 0.1247670000
8 2.2922270000 -0.1073440000 0.6752950000
1 1.6596580000 0.6549290000 0.4435320000
1 2.6286120000 -2.0415430000 0.3864950000

Frequencies

41.121	362.954	1007.6	1443.02	3118.99
66.8329	416.317	1023.44	1496.3	3150.78
95.3438	531.628	1047.83	1506.08	3170.65
124.477	689.144	1086.64	1510.95	3189.57
160.698	705.634	1098.91	1561.03	3300.94
167.85	720.921	1263.45	1655.86	
247.41	867.471	1287.65	1730.28	
275.081	914.749	1393.02	2784.3	
291.264	950.026	1409.2	3055.85	
320.691	1005.23	1420.76	3061.66	

syn-trans-MACR-oxide…FA

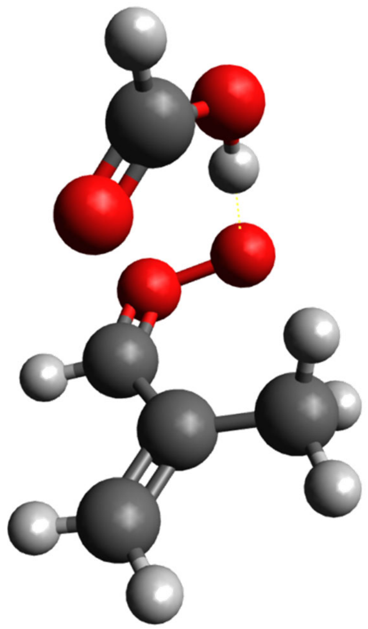

Coordinates
6 -1.2546950000 0.0107510000 1.5839420000
6 -1.5966550000 -0.1739430000 0.1360660000
6 -0.9088080000 0.4788580000 -0.9517220000
8 -0.0142910000 1.3703800000 -0.9421250000
8 0.5481020000 1.7376620000 0.2849720000
1 -1.2185010000 0.2536020000 -1.9652380000
6 -2.6042380000 -0.9677300000 -0.2722990000
1 -2.8341670000 -1.1005200000 -1.3195400000
1 -3.2120280000 -1.5064790000 0.4391420000
1 -1.9553140000 -0.5562180000 2.1935850000
1 -0.2450550000 -0.3368970000 1.7816990000
1 -1.2875600000 1.0590500000 1.8623920000
8 1.0143890000 -1.3745900000 -0.5896950000
6 2.0779760000 -1.1237960000 -0.0525660000
8 2.4395060000 0.0160400000 0.4863700000
1 1.6958120000 0.7183100000 0.3944610000
1 2.8736880000 -1.8716250000 0.0367910000

Frequencies

35.5047	353.3	1007.72	1460.11	3155.64
63.8316	435.407	1048.88	1484.89	3166.23
90.2518	538.62	1070.77	1501.5	3169.56
111.118	708.873	1081.75	1526.52	3190.08
168.815	721.277	1131.32	1573.59	3261.84
210.928	722.455	1270.01	1656.19	
241.016	832.528	1356.93	1730.6	
267.49	865.389	1388.39	2621.39	
296.385	941.646	1406.16	3054.42	
346.959	983.406	1428.04	3076.19	

Product Wells

Dioxole…FA

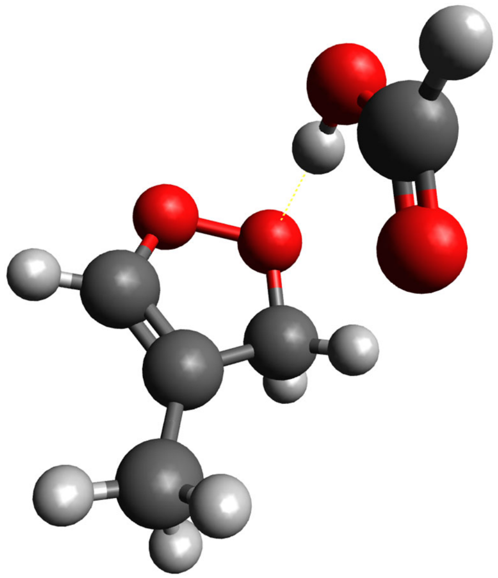

Coordinates
1 -1.8386050000 -0.8199450000 -1.8833160000
6 -1.4431790000 -0.6622050000 -0.8935850000
6 -1.5053100000 0.3844520000 -0.0835300000
6 -0.8211080000 -0.0235450000 1.1892950000
8 -0.7741970000 -1.7537030000 -0.4018890000
8 -0.1388090000 -1.2488630000 0.8409500000
6 -2.1274540000 1.7174420000 -0.2956630000
1 -1.5281710000 -0.2173650000 2.0020570000
1 -0.0502820000 0.6793910000 1.5009950000
1 1.4590010000 -0.8972250000 0.1137940000
8 2.3070030000 -0.6924850000 -0.3447690000
6 2.5209350000 0.6180120000 -0.3072850000
8 1.8090290000 1.4425430000 0.2151170000
1 3.4535560000 0.8630040000 -0.8252140000
1 -2.6021560000 1.7785540000 -1.2722140000
1 -1.3757890000 2.5048620000 -0.2291650000
1 -2.8850670000 1.9238510000 0.4623950000

Frequencies

26.3409	462.168	1032.79	1432.64	3095.95
34.9321	601.819	1076.57	1437.44	3113.12
76.0208	680.078	1085.47	1492.87	3136.42
135.524	768.37	1102.39	1508.78	3259.24
170.161	786.927	1202.95	1515.05	3408.31
195.658	821.269	1214.18	1738.17	
198.079	908.665	1251.52	1780.73	
243.679	924.151	1298.81	3013.99	
283.95	958.149	1375.01	3043.23	
304.641	1018.41	1386.23	3068.46	

Saddle Points

MACR-oxide *cis/trans* interconversion

anti-trans-MACR-oxide = *anti-cis*-MACR-oxide

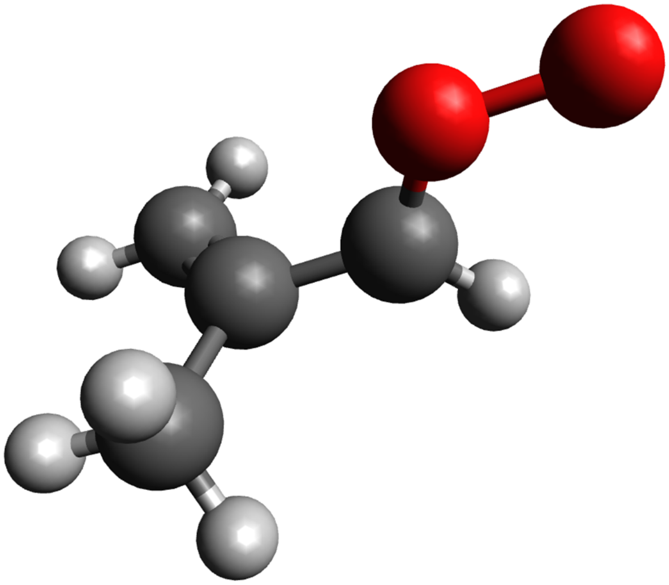

Coordinates
6 1.6092210000 1.2810260000 -0.1076060000
1 1.0940510000 2.2231800000 0.0060700000
1 2.6508580000 1.3127690000 -0.3942630000
6 0.9909840000 0.1184330000 0.0918290000
6 1.6531040000 -1.2239990000 -0.0538900000
1 2.7014560000 -1.1101110000 -0.3193980000
1 1.5882120000 -1.7941650000 0.8737120000
1 1.1551740000 -1.8100140000 -0.8267380000
6 -0.4253200000 0.1008020000 0.5045430000
1 -0.7784990000 0.1404460000 1.5297190000
8 -1.3150780000 -0.0149520000 -0.3960700000
8 -2.6073200000 -0.0625080000 -0.0387250000

Frequencies

-160.096	896.988	1471.25
153.056	966.841	1490.65
189.662	1000.33	1507.24
219.582	1023.9	1703.32
357.686	1040.42	3048.26
436.049	1088.65	3107.1
490.781	1276.95	3141.8
582.86	1327.85	3162.06
728.226	1419.04	3164.31
871.139	1439.36	3254.69

syn-trans-MACR-oxide = *syn-cis*-MACR-oxide

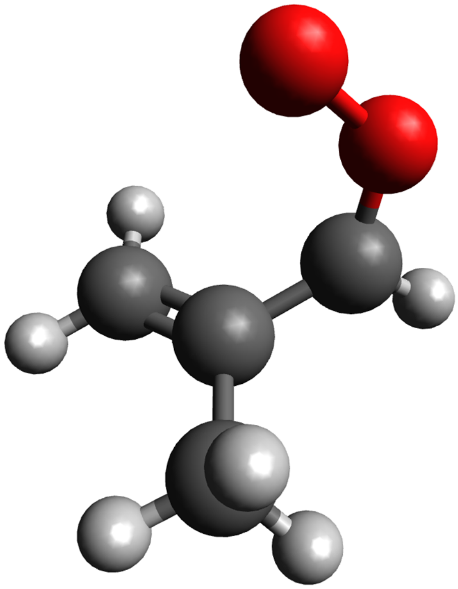

Coordinates
6 1.0987730000 1.4130990000 0.1035280000
1 0.4329200000 2.1633620000 0.5022950000
1 2.0079140000 1.7516560000 -0.3724360000
6 0.8093630000 0.1163770000 0.1730550000
6 1.6672970000 -0.9790280000 -0.3962120000
1 2.5773140000 -0.5667570000 -0.8256920000
1 1.9412140000 -1.7081860000 0.3674640000
1 1.1212970000 -1.5096310000 -1.1767190000
6 -0.4163940000 -0.3314480000 0.8469450000
1 -0.4292230000 -0.7904950000 1.8267920000
8 -1.5803740000 -0.2881580000 0.3238710000
8 -1.7453350000 0.2064150000 -0.9095710000

Frequencies

-103.553	910.198	1483.01
180.848	952.808	1494.57
201.495	980.392	1508.86
236.979	992.31	1706.1
386.677	1031.25	3046.69
427.726	1084.27	3106.06
549.602	1278.49	3140.73
659.366	1328.18	3166.43
737.322	1415.5	3195.21
777.507	1451.43	3257.29

MACR-oxide + FA 1,4-Addition (TSa)

anti-trans-MACR-oxide…FA = *anti-trans*-HPMAF

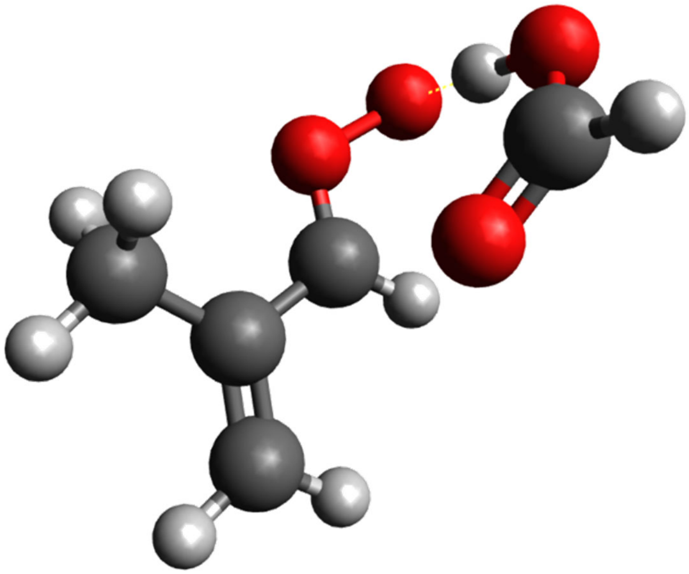

Coordinates
6 -2.2233470000 0.2795650000 1.2867650000
6 -1.7835920000 -0.0860100000 -0.0965160000
6 -0.5104380000 0.4097840000 -0.5699660000
8 0.0612030000 1.3304860000 0.0860120000
8 1.3091040000 1.7228130000 -0.4823810000
1 -0.1186560000 0.1671310000 -1.5461890000
6 -2.4786330000 -0.8549660000 -0.9442700000
1 -2.0899580000 -1.1038230000 -1.9209620000
1 -3.4428110000 -1.2587350000 -0.6733000000
1 -1.5085390000 -0.1004540000 2.0158070000
1 -2.2677640000 1.3603920000 1.4106930000
1 -3.2019180000 -0.1413130000 1.5000220000
8 0.7569100000 -1.4062490000 0.0273700000
6 1.9577830000 -1.2707800000 0.2818160000
8 2.6544010000 -0.1888980000 0.2393930000
1 2.0625260000 0.6912230000 -0.0820650000
1 2.5435410000 -2.1452000000 0.5858600000

Frequencies

-184.019	400.053	1007.31	1436.12	3126.69
55.2501	475.057	1065.39	1460.08	3155.14
66.5782	535.047	1067.78	1492.45	3170.79
125.46	566.525	1082.57	1504.16	3230.77
176.622	722.021	1226.8	1545.88	3262.94
189.435	753.328	1262.74	1655.16	
208.23	873.128	1318.47	1687.9	
257.123	887.941	1341.3	1780.5	
342.958	977.06	1396.81	3053.15	
372.216	992.604	1426.01	3064.15	

syn-cis-MACR-oxide…FA = *syn-cis*-HPMAF

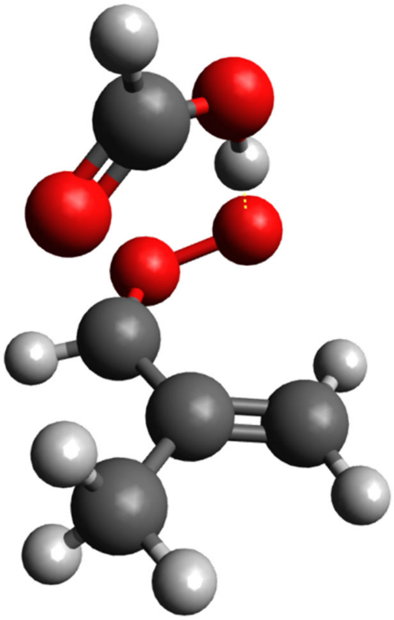

Coordinates
6 2.3427290000 1.0926430000 -0.0272690000
6 1.4290370000 -0.0880690000 0.1594210000
6 0.5346920000 -0.3185340000 -0.9637330000
8 -0.3681550000 -1.1989380000 -1.1376740000
8 -0.9171720000 -1.7763670000 0.0404170000
1 0.7897040000 0.1451230000 -1.9090330000
6 1.4915760000 -0.8998040000 1.2253830000
1 0.8091800000 -1.7224450000 1.3439100000
1 2.2517400000 -0.7289690000 1.9749140000
1 3.0238980000 1.1822020000 0.8144270000
1 2.9361940000 0.9955490000 -0.9371740000
1 1.7590400000 2.0077640000 -0.1082190000
8 -0.7032770000 1.4884940000 -0.4883430000
6 -1.6552170000 1.3261690000 0.2851480000
8 -2.0907820000 0.2295490000 0.7950240000
1 -1.5626600000 -0.6979810000 0.4678040000
1 -2.2289080000 2.2024200000 0.6042740000

Frequencies

-316.776	396.74	1017.41	1423.95	3121.38
55.7111	424.727	1044.57	1447.42	3153.23
78.9853	523.492	1061.95	1494.64	3172.97
151.582	681.495	1088.01	1512.39	3191.33
159.44	701.332	1156.58	1568.83	3297.64
211.42	763.756	1230.13	1654.15	
286.654	847.27	1286.03	1671.5	
299.621	914.897	1341.83	1773.22	
339.581	969.402	1387.73	3056.22	
370.987	1000.87	1415.17	3060.22	

syn-trans-MACR-oxide…FA = *syn-trans*-HPMAF

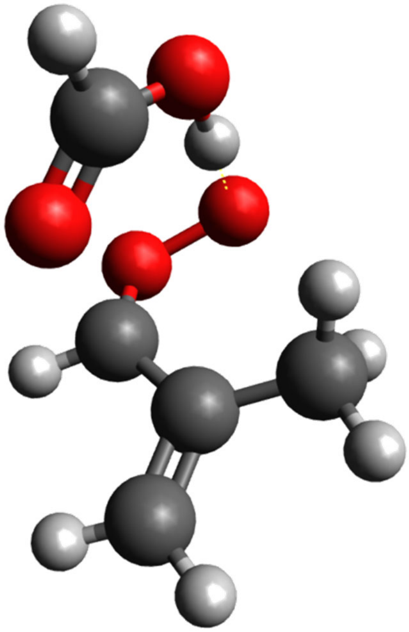

Coordinates
6 -1.2567360000 0.4120040000 1.5298760000
6 -1.4930120000 -0.0992950000 0.1406730000
6 -0.6483170000 0.2108440000 -1.0003110000
8 0.1777240000 1.1589350000 -1.1739410000
8 0.6831160000 1.7676300000 0.0112880000
1 -0.8900110000 -0.2648040000 -1.9423430000
6 -2.5416400000 -0.8783650000 -0.1717880000
1 -2.6934820000 -1.2542920000 -1.1730750000
1 -3.2694560000 -1.1565050000 0.5757910000
1 -2.0400010000 0.0400340000 2.1869740000
1 -0.2920690000 0.0777940000 1.9033480000
1 -1.2430980000 1.4969520000 1.5504070000
8 0.8248860000 -1.4512370000 -0.4826920000
6 1.8302430000 -1.1854510000 0.1833470000
8 2.1946380000 -0.0395090000 0.6456960000
1 1.5157300000 0.8069160000 0.3637800000
1 2.5262350000 -1.9910610000 0.4415290000

Frequencies

-282.031	397.719	1006.9	1430.87	3148.11
57.8743	441.972	1062.4	1463.06	3163.88
71.8933	525.359	1067.27	1486.79	3169.81
132.463	693.214	1084.79	1504.62	3200.16
204.744	728.265	1137.08	1574.93	3260.92
231.886	760.324	1256.82	1658.8	
272.249	830.985	1335.37	1667.41	
285.667	865.198	1350.16	1764.96	
343.809	954.998	1380.62	3053.22	
366.064	983.565	1414.6	3074.32	

MACR-oxide, Dioxole Formation (TSd)

syn-cis-MACR-oxide = Dioxole

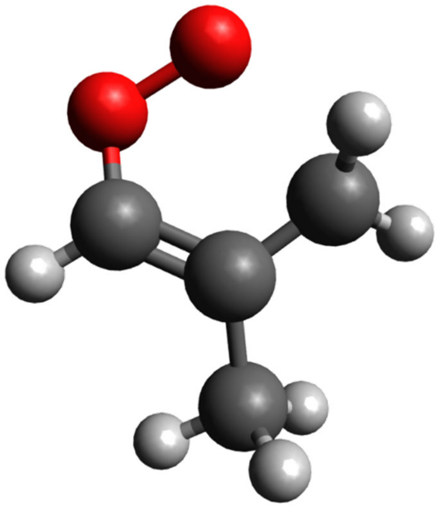

Coordinates
1 -0.0808780000 -2.0361170000 -0.1935480000
6 0.1811600000 -0.9883890000 -0.1861310000
6 -0.7163440000 0.0553410000 -0.1565180000
6 -0.2291240000 1.3648690000 -0.0499970000
8 1.4420110000 -0.7910140000 0.1332480000
8 1.7778130000 0.5408270000 0.0716440000
6 -2.1729720000 -0.2477100000 0.0987930000
1 -0.8175570000 2.0477150000 0.5633990000
1 0.4177060000 1.8311810000 -0.7630170000
1 -2.7970010000 0.0564450000 -0.7399510000
1 -2.5153510000 0.3042150000 0.9755330000
1 -2.3418350000 -1.3066130000 0.2815660000

Frequencies

-467.325	931.041	1491.09
138.397	965.047	1509.17
173.471	1008.11	1543.1
332.282	1018.48	1561.59
384.304	1057.26	3049.66
475.879	1088.35	3086.1
541.913	1245.89	3109.55
714.295	1273.84	3133.83
769.694	1418.37	3223.7
871.362	1424.94	3311.78

MACR-oxide + FA, Dioxole Formation from Spectator Catalysis (TSdc)

syn-cis-MACR-oxide…FA = Dioxole…FA

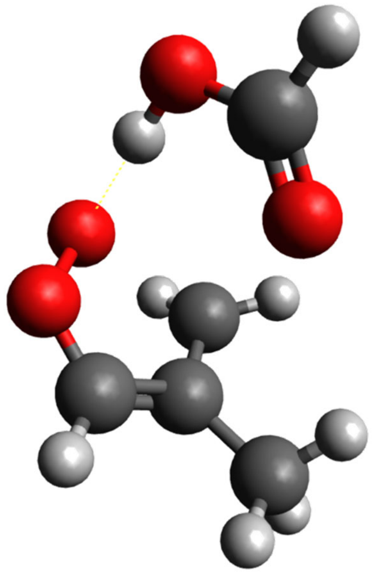

Coordinates
1 1.6718880000 -0.6239290000 -1.8866430000
6 1.2988650000 -0.7224080000 -0.8774900000
6 1.6366920000 0.0992550000 0.1717000000
6 1.0130950000 -0.1086980000 1.4035090000
8 0.2322250000 -1.4863970000 -0.8280970000
8 -0.2444570000 -1.6191160000 0.4794390000
6 2.3110630000 1.4134040000 -0.1401600000
1 0.7432030000 0.7799070000 1.9704850000
1 1.0815340000 -1.0202200000 1.9613780000
1 -1.6967630000 -0.7856250000 0.2895250000
8 -2.5440420000 -0.2722920000 0.1559050000
6 -2.2772160000 0.9844100000 -0.1592090000
8 -1.1804770000 1.4835970000 -0.2765680000
1 -3.2078970000 1.5442380000 -0.3070450000
1 2.6215860000 1.4617350000 -1.1813020000
1 3.1851450000 1.5793170000 0.4852190000
1 1.6003220000 2.2224580000 0.0328610000

Frequencies

-449.862	381.651	1021.09	1421.02	3124.1
31.7145	480.765	1045.17	1447.29	3131.92
59.5814	525.028	1060.63	1494.07	3140.85
66.6323	689.356	1095.49	1509.84	3223.1
120.85	720.148	1100.29	1551.05	3304.82
134.404	773.021	1227.82	1565.27	
168.571	887.635	1256.17	1767.71	
216.376	925.938	1278.47	3043.29	
251.806	939.677	1398.25	3055.89	
325.496	1010.8	1418.64	3115.17	

Products

HPMAF

anti-trans-HPMAF

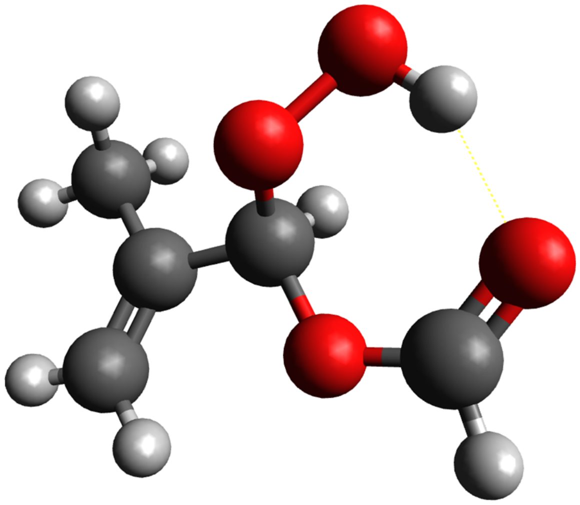

Coordinates
6 0.1664670000 0.0342290000 0.2169030000
6 1.6307850000 -0.2823040000 0.0733260000
6 2.0744730000 -1.4667780000 -0.3366110000
1 1.4031950000 -2.2722910000 -0.5835420000
1 3.1353120000 -1.6476330000 -0.4307370000
6 2.5264030000 0.8683930000 0.4251920000
1 2.3183830000 1.7256570000 -0.2131110000
1 2.3626490000 1.1889150000 1.4557650000
1 3.5711860000 0.5905010000 0.3144790000
1 -0.0761020000 0.4094320000 1.2103960000
8 -0.5883420000 -1.1931630000 0.0018380000
6 -1.9143490000 -1.1640380000 0.0967810000
8 -2.6151310000 -0.1987960000 0.2927400000
1 -2.3072990000 -2.1754780000 -0.0320850000
8 -0.1615100000 1.0110930000 -0.7246890000
8 -1.0652810000 1.9821950000 -0.1370800000
1 -1.8678760000 1.4332690000 -0.0371870000

Frequencies

47.4744	495.462	1013.34	1422.75	3111.97
71.9752	521.407	1041.26	1458.29	3148.5
140.437	579.03	1052.69	1489.01	3178.29
168.995	611.558	1074.75	1491.48	3274.7
240.478	732.501	1088.53	1509.89	3568.61
252.614	790.853	1228.63	1722.39	
256.082	900.805	1274.55	1760.06	
301.737	924.164	1356.07	3049.79	
371.412	952.033	1390.23	3091.85	
397.031	963.357	1417.17	3093.45	

anti-trans-HPMAF Geometry 2

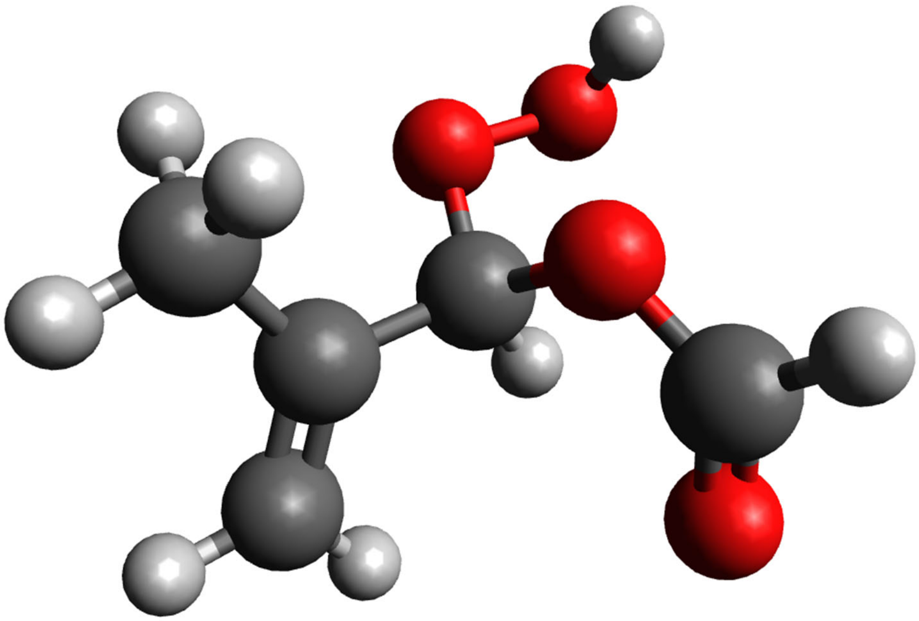

Coordinates
6 0.0108200000 0.2853950000 -0.2937400000
6 -1.3658580000 -0.2956980000 -0.1613990000
6 -1.7631330000 -1.1988200000 -1.0542410000
1 -1.1219960000 -1.5063910000 -1.8670430000
1 -2.7366140000 -1.6624130000 -0.9830390000
6 -2.1898910000 0.1630340000 1.0039460000
1 -3.1327220000 -0.3768420000 1.0428010000
1 -1.6555090000 0.0018580000 1.9401390000
1 -2.4000000000 1.2296120000 0.9334460000
1 0.5089900000 -0.0088630000 -1.2113290000
8 0.8401600000 -0.1423180000 0.8280440000
6 1.6192730000 -1.2194670000 0.6206530000
8 1.6739310000 -1.8782130000 -0.3819450000
1 2.2062430000 -1.4084980000 1.5252360000
8 -0.0974270000 1.6697540000 -0.1967220000
8 1.1916800000 2.2292560000 -0.5490900000
1 1.5975950000 2.2970310000 0.3261740000

Frequencies

47.1873	378.776	1005.02	1410.29	3143.98
55.7001	536.028	1044.45	1423.23	3149.56
128.283	554.706	1057.17	1469.93	3165.69
163.465	574.428	1084.38	1491.92	3254.38
178.412	726.066	1130.87	1511.31	3760.68
192.386	784.465	1191.62	1720.37	
249.421	871.451	1321.67	1785.13	
277.047	925.589	1358.23	3057.29	
313.973	933.845	1376.27	3064.67	
365.066	960.371	1387.47	3116.89	

syn-cis-HPMAF

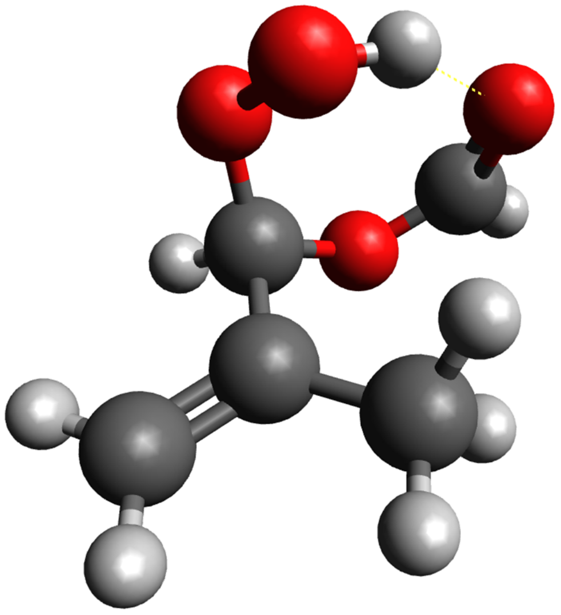

Coordinates
6 -0.3061810000 0.2905030000 -0.8449930000
6 -1.3856710000 -0.3352100000 0.0033650000
6 -2.6466560000 -0.0661400000 -0.3276230000
1 -2.8892400000 0.5647570000 -1.1709070000
1 -3.4715080000 -0.4563370000 0.2502610000
6 -1.0134930000 -1.2290670000 1.1470980000
1 -1.9104470000 -1.5930800000 1.6420480000
1 -0.4472100000 -2.0928750000 0.7982230000
1 -0.4009360000 -0.7026320000 1.8746130000
1 -0.6709130000 0.4848760000 -1.8496180000
8 0.7588940000 -0.7018330000 -1.1178770000
6 1.8505790000 -0.8429650000 -0.3774010000
8 2.1752570000 -0.2352660000 0.6177730000
1 2.4671660000 -1.6343050000 -0.8121090000
8 0.1771120000 1.5210500000 -0.4366290000
8 0.2897010000 1.5952280000 1.0047850000
1 1.1239080000 1.0934500000 1.1203950000

Frequencies

56.0857	483.693	995.395	1422.63	3132.62
74.6955	536.176	1047.23	1465.7	3152.12
169.678	639.493	1057.01	1494	3163.76
193.403	683.067	1082.5	1501.94	3250.1
224.893	747.521	1099.51	1512.56	3511.29
238.561	758.67	1210.77	1710.37	
286.937	854.113	1322.18	1750.76	
305.259	902.532	1356.71	3061.3	
365.6	915.407	1398.07	3083.16	
401.387	955.636	1418.66	3126.16	

syn-cis-HPMAF Geometry 2

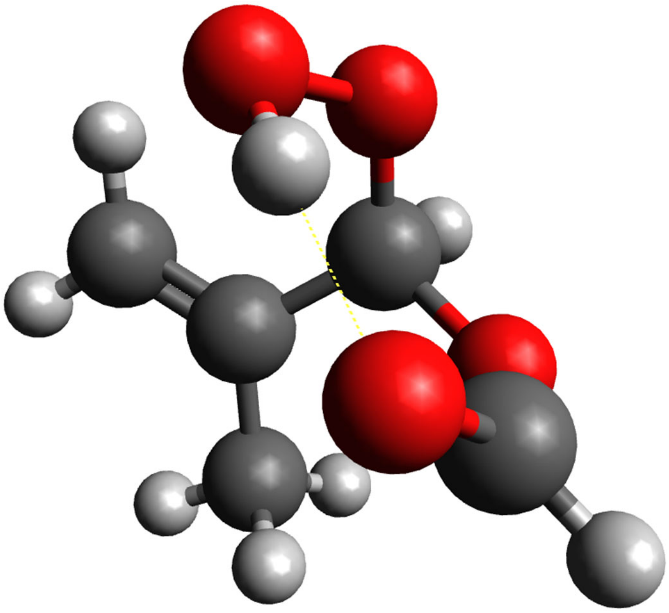

Coordinates
6 0.1815980000 0.1356200000 0.9073460000
6 1.3295820000 -0.1894060000 -0.0190590000
6 2.1002740000 0.7783000000 -0.5068930000
1 1.8724670000 1.8194320000 -0.3535210000
1 2.9756600000 0.5319540000 -1.0904210000
6 1.6221020000 -1.6471510000 -0.2294300000
1 0.8554190000 -2.1237660000 -0.8404950000
1 2.5769800000 -1.7663290000 -0.7347970000
1 1.6590260000 -2.1901860000 0.7155620000
1 0.5013540000 0.0399350000 1.9442360000
8 -0.8422840000 -0.9429950000 0.8663420000
6 -1.6717560000 -1.0751760000 -0.1573900000
8 -1.7981710000 -0.3377950000 -1.1094390000
1 -2.2686650000 -1.9815220000 -0.0253140000
8 -0.3737170000 1.3993620000 0.8720370000
8 -0.5394200000 1.9324940000 -0.4677630000
1 -1.1142950000 1.2488280000 -0.8721060000

Frequencies

45.2788	468.519	995.826	1426.86	3104.64
56.5167	532.95	1033.08	1459.73	3142.96
195.881	616.337	1054.75	1496.25	3176.86
206.538	654.725	1086.39	1506.13	3279.86
227.721	686.94	1108.34	1527.82	3499.13
256.518	773.242	1207.38	1714.56	
286.529	844.741	1290.57	1751.48	
308.202	900.471	1360.66	3050.64	
387.79	941.607	1407.44	3085.3	
394.853	964.925	1416.21	3098.06	

syn-trans-HPMAF

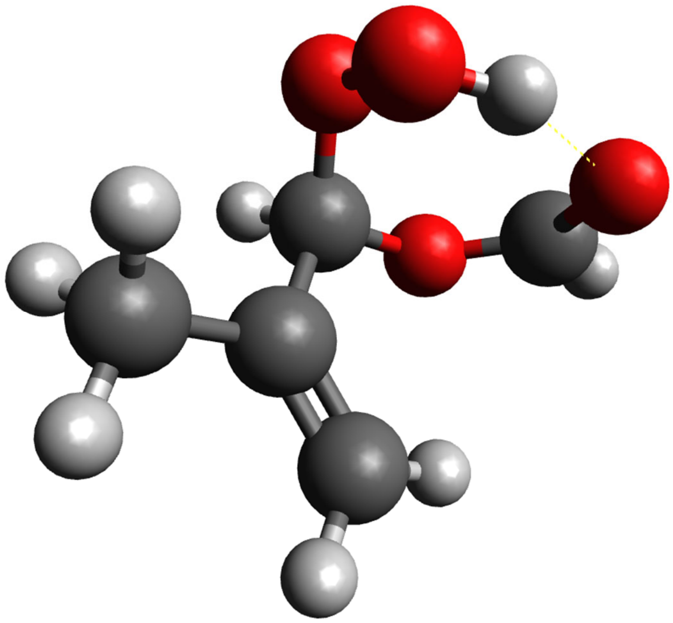

Coordinates
6 0.2002100000 0.0367680000 0.8776120000
6 1.2279820000 -0.5897170000 -0.0271250000
6 1.0149280000 -1.7355310000 -0.6656090000
1 0.0799480000 -2.2681630000 -0.6000530000
1 1.7910760000 -2.1724770000 -1.2763630000
6 2.5307290000 0.1533330000 -0.0697180000
1 2.3942660000 1.1349550000 -0.5173690000
1 2.9249690000 0.3035520000 0.9369810000
1 3.2662440000 -0.4020030000 -0.6459570000
1 0.5286210000 -0.0091010000 1.9160360000
8 -1.0384870000 -0.7480820000 0.9397480000
6 -2.0220010000 -0.6227810000 0.0555900000
8 -2.0971780000 0.1307520000 -0.8870730000
1 -2.8141650000 -1.3313190000 0.3114730000
8 -0.0145240000 1.3925840000 0.6712400000
8 0.0183530000 1.7218420000 -0.7393540000
1 -0.8273580000 1.3153530000 -1.0257370000

Frequencies

70.5295	480.974	993.335	1422.86	3120.91
83.915	514.586	1041.54	1458.38	3153.02
173.439	645.536	1051.63	1493.07	3177.99
194.137	675.637	1081	1509.1	3265.58
231.603	721.742	1094.09	1513.09	3493.24
239.065	777.979	1207.83	1721.93	
295.139	868.517	1282.23	1758.92	
302.108	914.457	1365.99	3052.19	
392.21	941.601	1402.24	3085.61	
422.015	947.353	1420.96	3091.25	

syn-trans-HPMAF Geometry 2

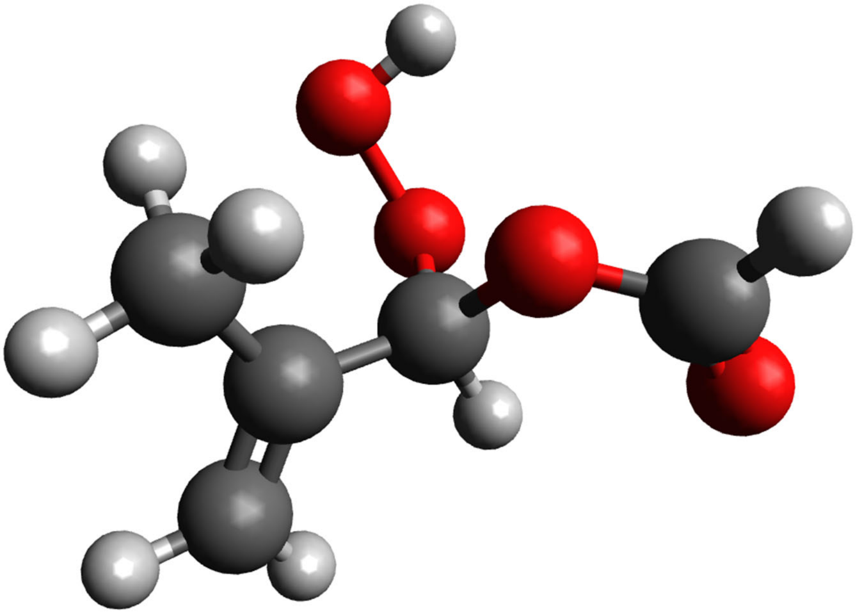

Coordinates
6 0.0595850000 -0.0775020000 -0.4114510000
6 -1.2788090000 -0.6587840000 -0.0412520000
6 -1.7920840000 -1.5754310000 -0.8605000000
1 -1.2733040000 -1.8910230000 -1.7544490000
1 -2.7523570000 -2.0287160000 -0.6612400000
6 -1.9629280000 -0.2137030000 1.2172780000
1 -2.8487800000 -0.8184190000 1.3972470000
1 -1.2959310000 -0.3095210000 2.0725520000
1 -2.2542270000 0.8316980000 1.1496910000
1 0.5616350000 -0.6869750000 -1.1581840000
8 0.8792370000 -0.0197170000 0.7763740000
6 2.1734420000 -0.3847560000 0.6390920000
8 2.7019380000 -0.7791450000 -0.3620080000
1 2.6706530000 -0.2713400000 1.6069690000
8 0.0467210000 1.1836430000 -1.0249010000
8 -0.6417930000 2.1306010000 -0.1669370000
1 0.1082410000 2.5123080000 0.3081920000

Frequencies

27.8594	405.562	993.625	1409.44	3136.39
38.2613	523.069	1045.63	1420.24	3147.17
129.71	549.124	1061.95	1468.86	3162.16
164.735	678.897	1077.65	1490.12	3247.97
187.775	734.916	1087.26	1516.57	3769.48
211.871	778.243	1166.43	1709.79	
229.695	866.936	1323.03	1794.28	
276.571	903.7	1356.51	3066.49	
286.844	953.35	1381.27	3074.83	
349.045	956.663	1387.45	3133.21	

anti-cis-HPMAF

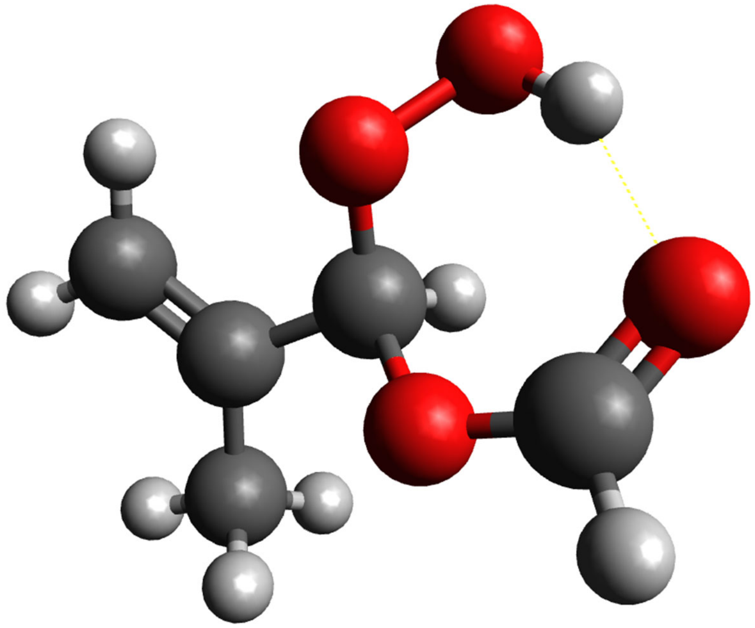

Coordinates
6 2.2657480000 1.2920740000 -0.4699020000
6 1.6554170000 -0.0098140000 -0.0414730000
6 0.1652370000 -0.1223080000 -0.2199360000
8 -0.3392730000 -1.2589570000 0.3891110000
8 -1.3561420000 -1.8539830000 -0.4547040000
1 -0.1274800000 -0.1112780000 -1.2689790000
6 2.3604030000 -1.0398110000 0.4178040000
1 1.8857150000 -1.9638640000 0.7043790000
1 3.4342340000 -0.9678460000 0.5106100000
1 3.3505280000 1.2331070000 -0.4348900000
1 1.9688950000 1.5466690000 -1.4891640000
1 1.9384020000 2.1085960000 0.1712000000
8 -0.3896120000 1.0988770000 0.3975790000
6 -1.6996810000 1.3081730000 0.3218900000
8 -2.5313160000 0.5757400000 -0.1629360000
1 -2.0609220000 -1.1810340000 -0.3723380000
1 -1.9413750000 2.2723590000 0.7764810000

Frequencies

56.4249	479.414	1015.75	1422.62	3111.73
80.3773	516.927	1030.02	1454.82	3146.16
142.372	580.931	1054.73	1493.28	3176.61
183.229	616.943	1089.22	1494.92	3274.25
233.955	726.582	1115.66	1508.44	3561.9
247.141	792.21	1219.6	1720.16	
263.309	870.387	1282.66	1754.78	
300.163	918.764	1351.74	3049.27	
372.568	957.135	1388.46	3088.07	
432.188	972.282	1414.04	3094.59	

anti-cis-HPMAF Geometry 2

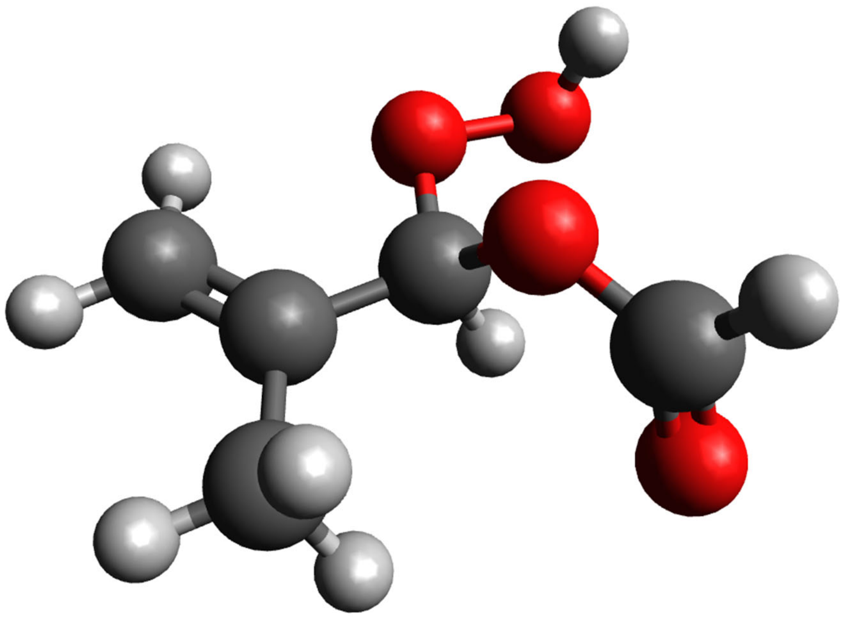

Coordinates
6 -0.0997050000 0.3275920000 -0.1685400000
6 -1.4478010000 -0.3320120000 -0.0586100000
6 -2.5690920000 0.3812800000 0.0050070000
1 -2.5593590000 1.4590150000 0.0084290000
1 -3.5278280000 -0.1147380000 0.0507170000
6 -1.4189710000 -1.8321030000 -0.0766610000
1 -0.8401990000 -2.2039320000 -0.9226940000
1 -0.9532040000 -2.2239590000 0.8274650000
1 -2.4283510000 -2.2295630000 -0.1419090000
1 0.3582970000 0.1638460000 -1.1414290000
8 0.8005980000 -0.2290880000 0.8319720000
6 1.7965220000 -1.0227230000 0.3972610000
8 1.9689620000 -1.4093300000 -0.7267370000
1 2.4354290000 -1.2780930000 1.2486330000
8 -0.2202060000 1.6849430000 0.0983540000
8 1.0111450000 2.3151720000 -0.3330730000
1 1.4655060000 2.4016420000 0.5159220000

Frequencies

43.8809	392.11	1016.27	1409.78	3116.08
49.9871	482.196	1029.36	1423.67	3145.86
121.349	551.834	1046.92	1459.75	3174.4
177.094	576.707	1086.39	1493.51	3270.36
193.265	713.442	1133.87	1511.27	3766.61
202.562	777.91	1190	1718.18	
252.107	904.526	1286.53	1784.78	
274.497	927.74	1381.32	3053.62	
341.887	951.089	1385.27	3066.62	
370.013	962.224	1396.09	3110.7	

References

1. Caravan, R. L.; Vansco, M. F.; Au, K.; Khan, M. A. H.; Li, Y.-L.; Winiberg, F. A. F.; Zuraski, K.; Lin, Y.-H.; Chao, W.; Trongsiriwat, N.; Walsh, P. J.; Osborn, D. L.; Percival, C. J.; Lin, J. J.-M.; Shallcross, D. E.; Sheps, L.; Klippenstein, S. J.; Taatjes, C. A.; Lester, M. I., Direct kinetic measurements and theoretical predictions of an isoprene-derived Criegee intermediate. *Proc. Natl. Acad. Sci.* **2020,** 117, 9733-9740.
2. Vereecken, L.; Novelli, A.; Taraborrelli, D., Unimolecular decay strongly limits the atmospheric impact of Criegee intermediates. *Phys. Chem. Chem. Phys.* **2017,** 19, 31599-31612.
3. Barber, V. P.; Pandit, S.; Green, A. M.; Trongsiriwat, N.; Walsh, P. J.; Klippenstein, S. J.; Lester, M. I., Four-Carbon Criegee Intermediate from Isoprene Ozonolysis: Methyl Vinyl Ketone Oxide Synthesis, Infrared Spectrum, and OH Production. *J. Am. Chem. Soc.* **2018,** 140, 10866-10880.
4. Vansco, M. F.; Caravan, R. L.; Pandit, S.; Zuraski, K.; Winiberg, F. A. F.; Au, K.; Bhagde, T.; Trongsiriwat, N.; Walsh, P. J.; Osborn, D. L.; Percival, C. J.; Klippenstein, S. J.; Taatjes, C. A.; Lester, M. I., Formic acid catalyzed isomerization and adduct formation of an isoprene-derived Criegee intermediate: experiment and theory. *Phys. Chem. Chem. Phys.* **2020,** 22, 26796.
